# Calibration Methods of Acoustic Emission Sensors

**DOI:** 10.3390/ma9070508

**Published:** 2016-06-24

**Authors:** Kanji Ono

**Affiliations:** Department of Materials Science and Engineering, University of California, Los Angeles (UCLA), Los Angeles, CA 90095, USA; ono@ucla.edu; Tel.: +1-310-825-5534

**Keywords:** acoustic emission, sensors, transducers, calibration, face-to-face, laser interferometry, Hill-Adams equation, tri-transducer method

## Abstract

This study examined outstanding issues of sensitivity calibration methods for ultrasonic and acoustic emission transducers and provides workable solutions based on physically measureable quantities, laser-based displacement measurement in particular. This leads to mutually consistent determination of transmitting and receiving sensitivities of sensors and transducers. Methods of circumventing problems of extraneous vibrations on free transmitters are used, giving the foundation for face-to-face calibration methods. Working on many ultrasonic and acoustic emission transducers, their receiving and transmitting sensitivities are found to be always different, while their ratios exhibit unexpected similarity. This behavior is attributed to monopolar pulse generation and bipolar received signals due to electrical charge transfer during elastic wave motion and reflection on the back face. This is verified through a quantitative piezoelectric sensing experiment. Displacement vs. velocity calibration terminology is clarified, redefining the “V/µbar” reference for contact sensor calibration. With demonstrated differences in the transmitting and receiving sensitivities of transducers, the requirement of the Hill-Adams equation invalidates the basic premise of the currently formulated reciprocity calibration methods for acoustic emission transducers. In addition, the measured reciprocity parameter for the case of through-transmission significantly deviates from the approximate theoretical prediction. It is demonstrated that three methods provide reliable sensor calibration results that are complimentary among them.

## 1. Introduction

**Figure 1:**
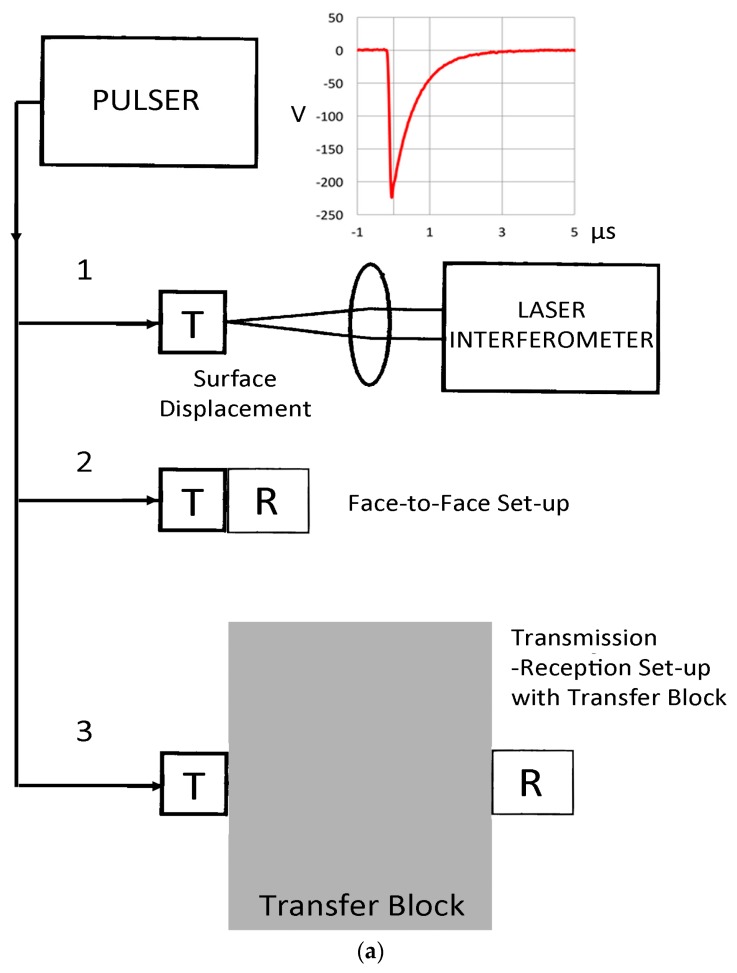

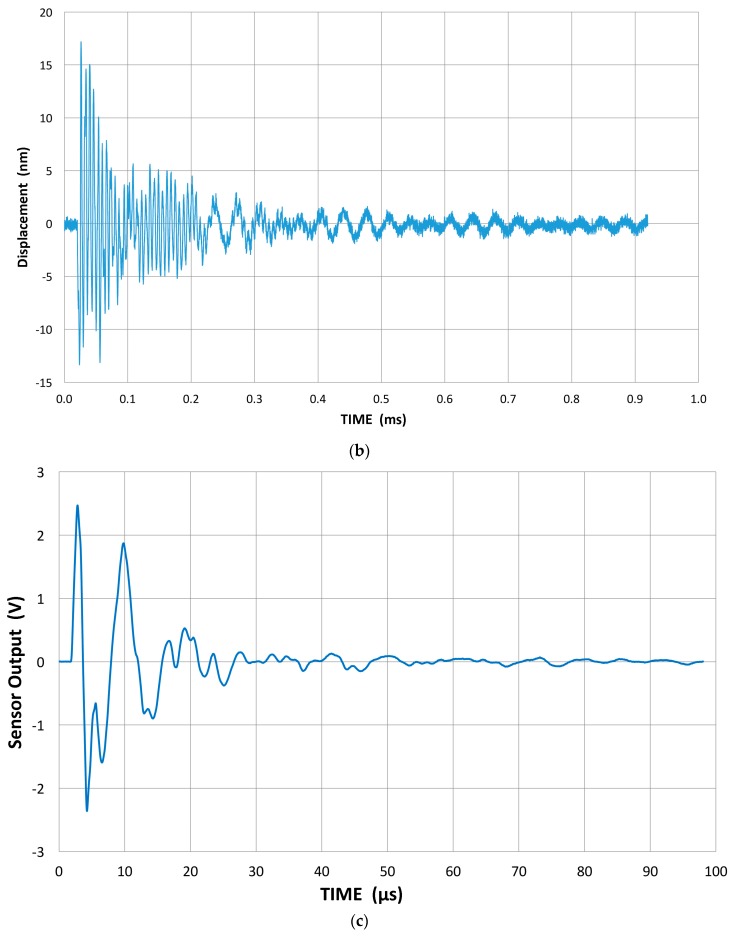
(**a**) Experimental arrangement. A pulser generates a short pulse, exciting a transmitter, T. Set-up 1: Surface displacement of T is measured with a laser interferometer. Set-up 2: Face-to-face arrangement of T and a receiver, R. Set-up 3: Transmission experiment with longitudinal waves passing through a transfer block; (**b**) Displacement vs. time of a pulse-excited resonant AE sensor (PAC R15), recorded by laser interferometry. The front face is exposed to air only; (**c**) Output of a broadband receiver (Olympus V101) coupled to the same R15 transmitter, excited in the same manner. The front face is coupled to the V101 transducer, terminated with 10 kΩ.

In ultrasonic testing (UT) and acoustic emission (AE) testing, transducers and sensors are essential components of a nondestructive test (NDT) system. The characterization of this component has been the subject of many studies over the years. Notable reviews on AE sensor calibration were provided by Sachse and Hsu, Hsu and Breckenridge and Hill [1,2,3]. Recently, three books on ultrasonic transducers appeared; one is by Schmerr and Song [4] and another edited by Vives [5]. Both books have analyzed ultrasonic transducer behavior from the systems approach, providing a good foundation of system modeling based on the electrical equivalent circuit representation. Using the reciprocity principle and reflector responses, transducers immersed in fluid have been characterized satisfactorily. The third book is by Sherman and Butler [6], which is directed toward underwater acoustics, but provides the most detailed discussion on piezoelectric sensing with an electroacoustic approach. In extending this lumped-parameter approach to cases of interest in NDT (or non-destructive evaluation, NDE), the need of the network analyzer presents an obstacle, since it is not commonly available in NDE laboratories. The other is the extensive use of contact transducers that often change their behavior from the air-loaded condition to a solid-loaded state. An example is shown in Figure 1. Figure 1a illustrates the three experimental methods used. A pulser generates a short pulse of a 200+ V peak with a few µs duration, which drives a transmitting transducer. In set-up 1, the surface normal displacement is detected by a laser interferometer at the center of the transmitter, T. In set-up 2, it is detected by a receiving sensor, R, which is acoustically coupled to T via a thin couplant layer. In set-up 3, T and R are separated by a solid transfer block. Figure 1b shows the waveform from set-up 1. This AE sensor is made from a piezoelectric disk with minimal damping and its transmission curve (displacement vs. time by laser interferometry) reverberates to 0.9+ ms upon its excitation by a few µs-long pulses (see the top of Figure 1a) with a persistent 30 kHz component. When it is in contact (so-called face-to-face arrangement, set-up 2) with a broadband receiver (Figure 1c), trailing oscillations essentially dissipate by 80 µs. Moffatt [7] discussed this front-face loading effect while developing rod-backed broadband sensors. It was also recognized in applying laser techniques to transducer calibration [8]. Such changes and other damping effects are difficult to predict theoretically and have not been treated satisfactorily. In some well-damped transducers, laser techniques provided satisfactory results, although radial resonance effects are more difficult to eliminate. In the present study, laser interferometry is utilized as the basis for transducer characterization. This was discussed extensively by, e.g., Scruby and Drain [8], but has so far not been widely used in NDE practice due to high cost. In UT transducer calibration, more advanced methods have appeared and the frequency range is extended to 20 MHz routinely [9] and to 100 MHz in the laboratory [10,11,12,13,14]. These include uses of direct measurement of particle velocity in water, time-delay spectroscopic methods, an optical multilayer hydrophone as a reference and pulse-echo with a reflector.

The reciprocity calibration methods have been well established for acoustic transducers and hydrophones. These also apply to some piezoelectric transducers. MacLean [15] started with the laws of reciprocity requiring the condition of reversibility. Let us denote the transmission output of the i-th transducer due to an electrical pulse as *T*_i_ = *t*_i_ × *V*_i_, where *t*_i_ is the transmit transfer function and *V*_i_ is the fast Fourier transform (FFT) spectrum of the pulse input. Here, the transducer output is measured in displacement. *R*_j_ is the receiving displacement sensitivity spectrum of the j-th transducer. With the above notation (without separating the input spectrum), the law of reciprocity for transducers i and j is

*T*_i_ × *R*_j_ = *T*_j_ × *R*_i_ (1)

which can also be stated as

*R*_i_/*T*_i_ = *R*_j_/*T*_j_. (2)

This ratio is equivalent to the reciprocity parameter as defined by MacLean [15]. In using the reciprocity approach, one must first establish the reciprocity of a transducer. While this reversibility requirement for the reciprocity calibration was in MacLean [15] and Foldy and Primakoff [16], it has become necessary only for an auxiliary transducer.

**Figure 2:**
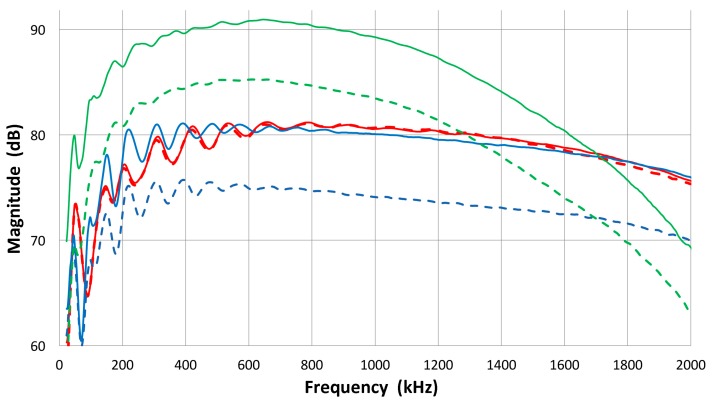
FFT magnitude spectra of received signals in transmission-reception experiments. Three pairs of transducers used. Red solid/dash curves: AET FC500-1 and -2. Blue solid/dash curves: Olympus V104-NDT Systems C16. Green solid/dash curves: Olympus V103-C16.

Attempts [17,18] have been made to extend this approach to contact piezoelectric transducers that are often damped for wideband applications. In such instances, however, the assumption of the reciprocal behavior of transducers as transmitter and receiver can no longer hold universally. Simple experiments reveal the breakdown of reciprocity. Three pairs of well-damped ultrasonic transducers show reciprocal and non-reciprocal behavior in a transmission-reception experiment using set-up 2, as seen in Figure 2. Results are given in terms of FFT magnitude spectra of received signals. Each pair was directly coupled and one was excited by a short pulse and the other used as a receiver; for transducers i and j, the FFT spectrum of the output voltage is defined as *E*˚_ij_. Next, the roles are switched, yielding *E*˚_ji_, and the pairs of *E*˚_ij_ and *E˚*_ji_ are plotted in Figure 2. One pair shows almost identical waveforms (not shown) and their FFT spectra (as overlapping red curves) agree closely. This pair consists of two AET FC500 (2.25 MHz, 19 mm diameter) with similar design and reciprocal behavior is expected. The second and third pairs (green and blue curves) have spectra differing by 4.5 to 7 dB. These pairs are between the NDT Systems C16 (2.25 MHz, 13 mm diameter) and the Olympus V103 (1 MHz, 13 mm) or V104 (2.25 MHz, 25 mm). Thus, changes in the center frequency or element size are a possible cause for the reciprocity breakdown. The damping design may also be different since spectral curves are of completely different shapes in some combinations. Results from 25+ pairs indicate that these are usually not reciprocal. The reciprocity behavior is found in less than a third of the cases, even when the reciprocity condition was allowed to break down over some frequency ranges. Typically, reciprocity requires nearly identical transducer design and specifications.

In the above FFT analysis, we used the FFT routine of Noesis software from Enviroacoustics, ver. 5.8. The sampling interval is kept at 2 ns and zero padding is used to keep the sample length of 262,144 points, giving the frequency step of 1.907 kHz. In most cases, a smoothing filter was applied to reduce noise, cutting off the low frequency response below 20 kHz.

Hill and Adams [19] showed that when the transmitting and receiving sensitivities of transducers are different, the ratio of the transmitting and receiving sensitivities for one of three transducers is required for the reciprocity equation expressing the receiving sensitivity. Following [19], for a directly coupled pair of transducers i and j, the following expression is obtained, ignoring the effect of the couplant layer between them:

*E*˚_ij_ = *T*_i_ × *R*_j_ = *t*_i_ × *V*_i_ × *R*_j_ (3)

When the pair is coupled through a transfer block with the propagating medium transfer function of *X*_ij_, it is written as

*E*_ij_ = *T*_i_ × *X*_ij_ × *R*_j_ = *t*_i_ × *V*_i_ × *X*_ij_ × *R*_j_, (3a)

where *X*_ij_ = *X*_ji_ = *X* is assumed in the reciprocity methods and *E*_ij_ = *E*˚_ij_ × *X*_ij_.

As is normally the case in reciprocity calibration, we select transducer pairs of 1 and 2, 1 and 3, and 2 and 3. Here transducer 3 is the auxiliary transducer and needs to be reversible and 2 is the target transducer of the calibration. Using the same designation, Equation (3) leads to

*E*˚_12_ = *T*_1_ × *R*_2_, *E*˚_13_ = *T*_1_ × *R*_3_, and *E*˚_32_ = *T*_3_ × *R*_2_. (4)

From these equations, *E*˚_12_/*E*˚_13_ = *R*_2_/*R*_3_, we get

*R*_2_ = (*E*˚_12_ × *R*_3_)/*E*˚_13_ and *R*_2_ = *E*˚_32_/*T*_3_. (5)

Combining, we have the following equation for directly coupled pairs

*R*_2_ = {(*E*˚_12_ × *E*˚_32_)/*E*˚_13_] (*R*_3_/*T*_3_)}^1/2^. (6)

Or for pairs coupled through the transfer block

*R*_2_ = {(*E*_12_ × *E*_32_/(*X* × *E*_13_)] (*R*_3_/*T*_3_)}^1/2^. (6a)

It is appropriate to name this Equation (6a) as the Hill-Adams equation. Here, there are six unknowns of *T* and *R* for transducers 1 to 3, but *T*_2_ was unused and *T*_1_ cancels out in Equation (5). To obtain *R*_2_, three *E*_ij_ values and *T*_3_ and *R*_3_ must be supplied from the experiment. In general cases of differing transmitting and receiving sensitivities, Hill and Adams [19] showed that the reciprocity calibration method is invalid without using the independently determined transmitting and receiving sensitivities of an auxiliary transducer. This condition of different transmitting and receiving sensitivities prevails in many piezoelectric transducers, thus posing a doubt as to the validity of the so-called “absolute reciprocity calibration” advocated by Hatano [17,18] and followed by others [20,21,22,23]. These researchers have all ignored the invalidity problem raised by Hill and Adams [19].

This problem can be overcome when transmitting sensitivities are obtained using laser interferometry. This also provides receiving sensitivities as will be shown later. Using the laser-based measurement of *T*_3_ and *R*_3_, the formalism of reciprocity calibration, namely Equation (6), can be utilized in conjunction with the face-to-face procedure of set-up 2.

The potential for an alternate approach should be noted here: by conducting the bi-directional transmission-reception experiments for all the possible combinations of a set of transducers, one can in principle use the genetic algorithm optimization procedures to separate the transmitting and receiving sensitivities (see, e.g., [24,25]). When such a software is written, the needs for laser interferometry can be minimized.

ASTM standards for ultrasonic transducer calibration are limited to getting the flaw position and flaw size using of calibration blocks [26,27]. Transducer manufacturers typically provide a back-echo waveform and its spectrum, but not the transmitting or receiving sensitivities. For conventional ultrasonic tests, this is apparently adequate, since costly procedures are required to get other desirable transducer characteristics such as beam spread, focusing, and angular sensitivity as well as the spectral sensitivities mentioned above. However, it is still advantageous to have both transmitting and receiving sensitivities for many advanced applications. Two ASTM standards do exist for the primary and secondary calibration of AE sensors [28,29]. These are based on seismic pulse on a large transfer block, developed at National Institute of Standards and Technology (NIST) and based on the work of Breckenridge et al. [30], and are used for the calibration of the surface wave sensitivity. Burks and Hamstad [31,32] reexamined the NIST procedures and suggested revisions. One of them is to use measured displacement rather than analytical calculation since the capillary break source is elliptical rather than a point. At present, however, no standards organization provides a calibration service based on these two standards. Also, no ASTM standard exists for AE sensor calibration for normal incidence waves.

AE sensor manufacturers typically provide a sensitivity curve based on face-to-face calibration. This calibration procedure has been treated as proprietary information and described only inadequately. Calibration curves are usually in reference to the reference level of 1 V/µbar, but this reference remains undefined. The ASTM E976 standard guide [33] is often referred to as the basis, but E976 specifically excludes the face-to-face procedure. This method does benefit from ease of set-up, reasonably good repeatability and the ability to handle long-duration signals of high-sensitivity (undamped or minimally damped) AE sensors. Commonly used AE sensors reverberate beyond 1 ms when free and often over 200 µs even coupled to a metal block, enlarging the needed size of a transfer block to inconvenient sizes of over 1 m. Recent papers [34,35] indicate renewed interest in this method and we will consider various aspects of the face-to-face procedures. However, it is suspected that the calibration unit is misconstrued in [34] regarding the velocity calibration, and their results are about 140 dB off the manufacturer’s calibration of the PAC R15 sensor. In sum, currently no applicable standards for calibrating ultrasonic transducers and AE sensors exist. This is indeed a dire situation for NDE professionals.

Before embarking on the main topics, some valuable early works on AE sensors should be brought up. Moffat et al. [7] developed rod-backed wideband velocity-sensitive sensors. These used 5 MHz PZT disks, backed by impedance-matched brass rods. Their 3.2-mm-diameter sensor achieved a smooth spectral behavior from 20 to 700 kHz with a 10 dB decrease according to the calibration at NIST. Another worthy sensor design used a polyvinylidene difluoride (PVDF) foil sensor with a small aperture backing. Chang and Sun [36] showed one design with an aperture 1.5 mm in diameter, which provides a NIST-sensor-like displacement-sensitive sensor. With a rod backing, it gives velocity-sensitive sensors as in [7]. 

We attempt here to examine the inadequacy of sensitivity calibration methods for AE sensors available today and to provide workable solutions based on physically measureable quantities. We start with the laser-based calibration of transmitting devices. This is then used to calibrate receiving sensitivities of sensors/transducers. Methods of validating both characteristics in combination are introduced in order to circumvent problems of extraneous vibrations that are found in some transmitters without front-face loading. Effectiveness of the face-to-face calibration method is evaluated, especially in connection to the ill-defined “V/µbar” reference in reporting contact sensor calibration. Displacement vs. velocity calibration terminology is clarified. Finally, reciprocity calibration methods are critically examined since the commonly used methods are found invalid without independently measured auxiliary transducer sensitivities. In lieu of these invalidated reciprocity methods, a tri-transducer method is developed. This new method provides calibration results comparable to laser-based direct and indirect methods. 

## 2. Transducer Sensitivity Calibration—Transmission

The use of laser interferometry for transducer calibration is straightforward as discussed by Scruby [8], but the surface loading effects need to be considered as extraneous vibrations sometimes occur when the transducer front face is free, only facing air. Commercial interferometers of various design are now available, although their uses have been limited due to high cost. Notable interferometry works came from UK-NPL [11,37] for the determination of acoustic pressure in water for the validation of reciprocity calibration of hydrophones and for AE sensor calibration with a through-transmission block. We reported on getting transfer functions of AE transducers [38], and other calibration methods were explored elsewhere. The work of Goujon and Baboux [20] was a significant advance as they used a laser interferometer to verify the displacement of surface pulse and obtained the sensitivity for the surface-wave reception of PAC µ-80 sensors. It was comparable to the representative calibration curve published by the manufacturer (using the NIST procedure). Keprt and Benes [22] followed up this study and reported the surface wave–receiving sensitivity of PAC UT1000 sensors. They used two reciprocity methods and the NIST capillary break method and the results match each other well below 300 kHz and reasonably (10 dB) up to 1 MHz. In these two studies, however, key details are unavailable for the reciprocity methods, so their validity in view of the Hill-Adams analysis cannot be assessed.

**Figure 3:**
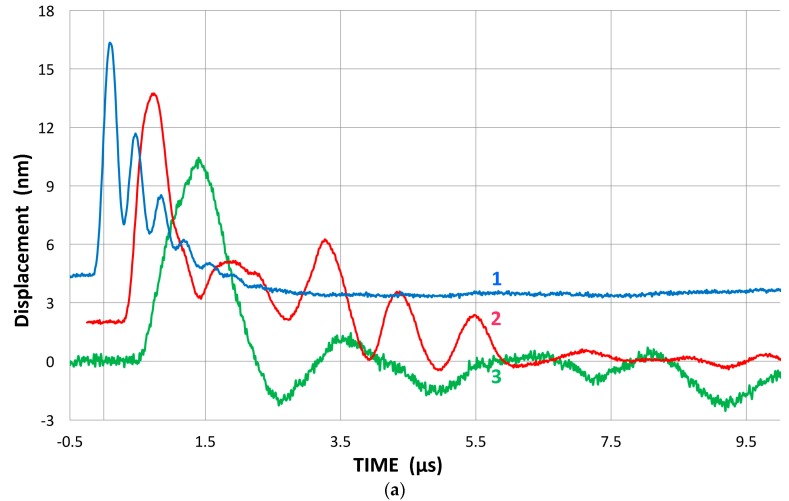

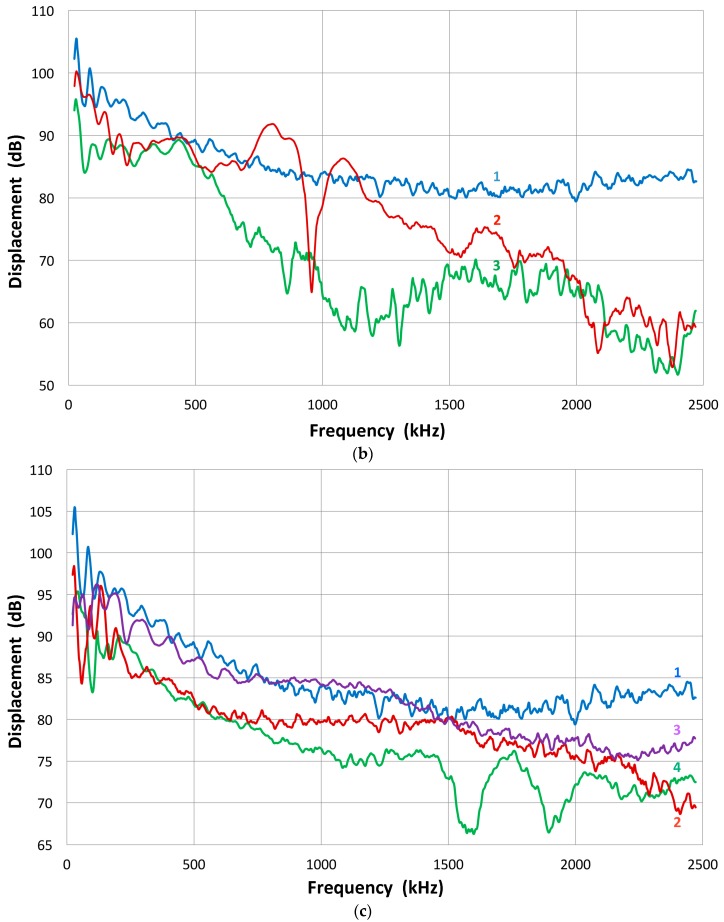
(**a**) Three typical displacement transmission curves for broadband ultrasonic transducers (Olympus V104 (1: blue curve), V103 (2: red) and V101 (3: green)); (**b**) Corresponding FFT magnitude spectra in dB scale against frequency. 1: blue curve-V104. 2: red with a sharp dip-V103. 3: green-V101; (**c**) Three more FFT magnitude spectra for FC500-1 (2: red curve, shifted down 5 dB) and -2 (3: purple) and NDT C16 (4: green, shifted down 10 dB). V104 (1: blue) also shown for comparison.

In our studies that have been directed toward normal incident waves, we have used a displacement-sensitive laser interferometer (Thales, LH140, Mach-Zehnder heterodyne type, 20 MHz bandwidth with a sensitivity of 0.1 V/nm at Aoyama Gakuin University, Sagamihara, Japan; Dr. H. Cho graciously conducted measurement), using set-up 1. Three typical transmission curves with a fast initial rise and slower decay with several oscillations are shown for broadband ultrasonic transducers (Olympus V101, V103 and V104; V101 is 0.5 MHz, 25 mm) in Figure 3a with their corresponding FFT magnitude in dB scale (Figure 3b). The peak displacement values are 10–12 nm and represent the out-of-plane displacement of the center region of the transmitter with a 100-times signal averaging. Except within 1 mm from the edge, the amplitude variation was less than 0.5 dB. The high voltage excitation pulse used was of a short rise time (0.2 µs) to the peak of 220 V nominal and decays to 10% in 1.5 µs or 3 µs to 0 (see Figure 1a). The FFT spectra of three more transducers (two FC500, NDT C16) are shown in Figure 3c. These are shifted in time and level as noted.

The observed displacement waveforms are basically of monopolar shape with trailing oscillations. This feature results from the forward radiation at the front face and the presence of the absorber behind the piezoelectric element, as predicted by Redwood and Kossoff [39,40]. The peak displacement value is approximately 50 pm/V. This displacement is about one half that of a typical lead-metaniobate piezoelectric element, which is presumed to be used in these transducers. Alternate elements of PZT ceramics are expected to produce displacements that are at least a few times larger. The other half of the piezoelectric displacement is radiated backward into the absorber and mostly damped. In addition, it is expected that a higher acoustic impedance of the sensor face material (usually alumina) causes a 2.3 dB reduction and also gives back reflection.

**Figure 4:**
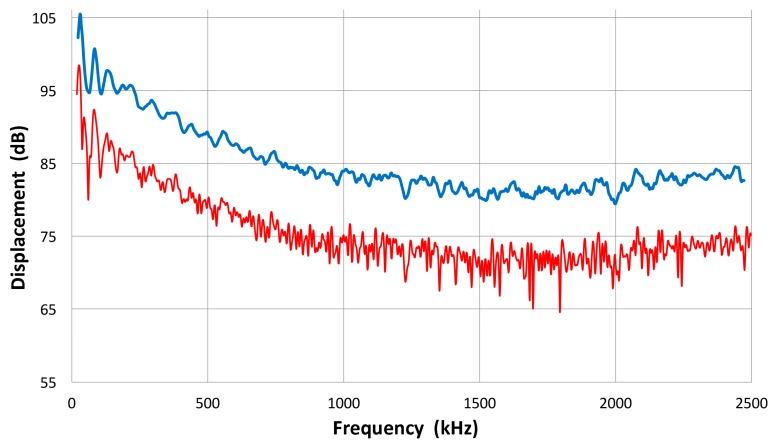
Effect of smoothing Savitzky-Golay filter on the V104 spectrum. Top with filter. Bottom—the original FFT spectrum, shifted down by 10 dB.

The FFT magnitude transmitting spectrum is relatively smooth for V104 (nominally 2.25 MHz center frequency), as can be seen in Figure 3b. The spectrum of V101 (0.5 MHz) exhibits a broad peak just below the nominal center frequency and lesser oscillations, while that of V103 (1 MHz) shows many peaks and a sharp dip just under 1 MHz, its center frequency. This figure shows that extraneous oscillations are indeed present from the lack of front-face loading of V103 (and V101 to a lesser extent). With face-to-face arrangements (set-up 2), these are absent since it was seen in Figure 2b that these transducers produced smooth spectral curves in combination with the NDT C16 (2.25 MHz) receiver. The latter (C16) as a transmitter also showed large oscillations above 1.4 MHz in the interferometry spectrum (Figure 3c), but Figure 2b also exhibited a smooth spectral curve in combination with V103 as a receiver. Thus, the front-face loading most effectively suppressed extraneous vibrations in these three transducers (V101, V103 and C16) used as transmitters. Two more transmitting spectral curves are shown in Figure 3c for two AET FC500 (2.25 MHz, 19 mm). These generate an output range similar to that of V104, but show additional oscillations overall, especially at low frequencies below 250 kHz. It appears that the low frequency oscillations come from radial resonance that was not suppressed adequately. From the comparison of these six transmitting spectral curves, we decided to use V104 as the reference transducer and use the remaining five for confirmation by avoiding the range where irregular changes are observed. The raw FFT spectral data contained noise and it was reduced by using the 25 point smoothing Savitzky-Golay algorithm [41]. Differences can be seen in Figure 4. The blue curve is after smoothing and the red one is the raw FFT magnitude spectrum, as obtained from FFT (shifted down 10 dB for visibility). The smoothed blue curve does match well and follows the middle of the noisy red curve.

**Figure 5:**
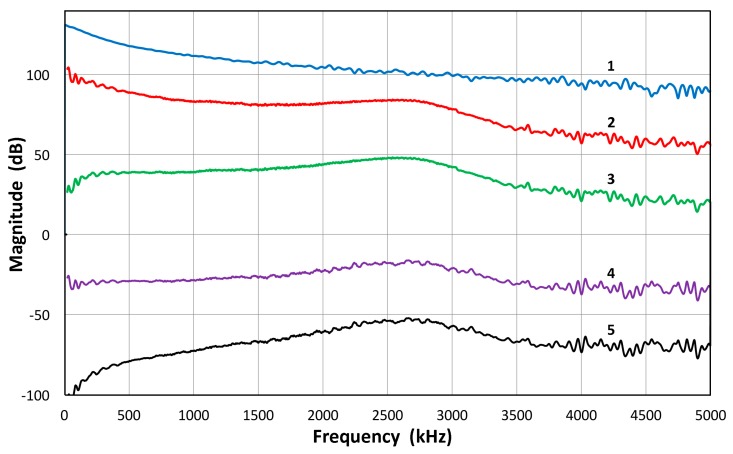
Transmission characteristics of V104 up to 5 MHz: 1 (blue): HV pulse spectrum in V; 2 (red): Displacement transmission spectrum including HV in nm; 3 (green): Velocity transmission spectrum including HV in m/s; 4 (purple): Displacement transmission spectrum excluding HV in nm/V; 5 (black): Velocity transmission spectrum excluding HV in m/sV.

The magnitude spectra in Figure 3b,c do include the spectrum of the high voltage (HV) electrical pulse, which is a slowly decreasing function with frequency. It varies only slightly (within ±1.5 dB) among most transducers used. The transmission characteristics of V104 are shown in Figure 5 up to 5 MHz. This higher range covers the frequency of interest in UT. The HV pulse spectrum for the V104 test is included as the top curve, marked 1. The displacement transmitting spectra of V104 are curves 2 and 4. The latter is corrected by subtracting the HV FFT spectrum, indicating much less frequency dependence of V104 transmission and showing a broad peak at 2.6 MHz. The value of Q is 1.7. By multiplying the angular frequency, these curves are converted to show the velocity response of V104 transmission, shown as curves 3 and 5, with or without the HV electrical pulse spectrum. It was confirmed that the discrete differentiation of the displacement waveform using the Savitzky-Golay algorithm [41] results in an identical magnitude spectrum as that due to 2 π*f* multiplication (*f* = frequency), shown here. 

Utilizing laser interferometry measurement (set-up 1), V104 is identified as a suitable reference transducer for providing the source of the displacement pulse. The displacement (and velocity) transmission characteristics will be used next for obtaining mutually consistent calibration of transducers using set-up 2.

## 3. Transducer Sensitivity Calibration—Reception

### 3.1. Direct Method

Once the transmitting sensitivity of a reference transducer is obtained, the receiving sensitivity of other transducers is determined by conducting the transmission-reception measurement in a face-to-face arrangement (set-up 2) of the reference against a transducer under test. This is the “Direct” method and gives the FFT magnitude spectral curves (with the unit of V), as shown previously in Figure 2, for the reversibility testing. The magnitude spectral curves represent the product of transmitting and receiving sensitivities (or the sum in terms of decibel values as we use them here). The receiving sensitivity of a transducer is determined by the face-to-face spectrum minus the transmitting sensitivity of the reference (V104) transducer (unit = nm). The 0 dB reference for the receiving sensitivity is 1 V/nm with frequency in kHz.

**Figure 6:**
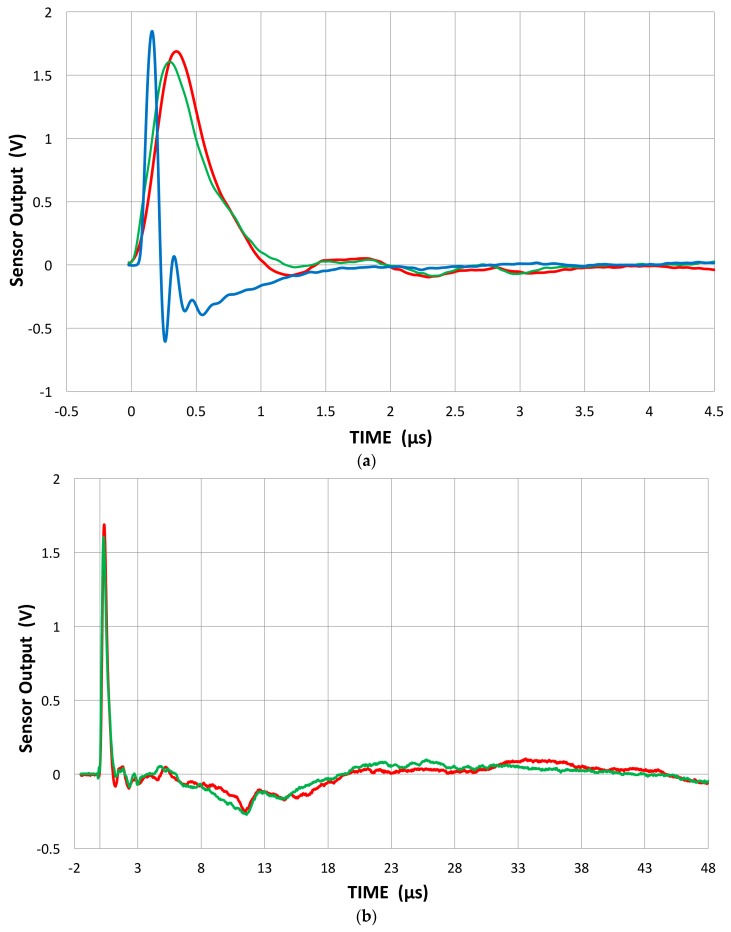

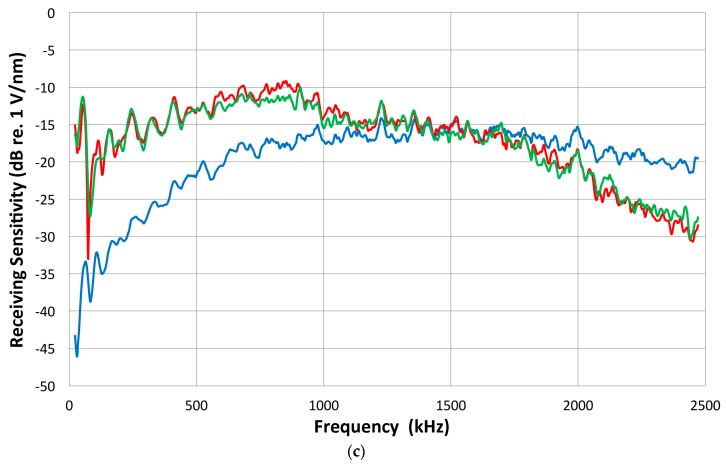
(**a**) Received waveforms of three conical sensors; Home-made (blue curve) and two KRN (red −35, green −60). High input impedance of 100 MΩ used; (**b**) Received waveforms of KRN sensors (red −35, green −60) to 48 µs; (**c**) Receiving sensitivities of the conical sensors (home-made: blue, K-35: red, K-60: green).

First, we examine the response of conical PZT-based sensors. One is home-made using the Proctor design [42] and two are KRN sensors of Harwell-Glaser design. Our own design used a Zn-4Al alloy (Zamak3) casting with a good (+2%) impedance matching to PZT-5A as the backing material [43]. Three receiver waveforms (Figure 6a,b) and their receiving sensitivities are shown in Figure 6c. The home-made sensor has a narrow initial pulse, with a partial reflection, and its signal diminished beyond 3 µs. The reflection was probably due to a thin insulation layer on the backing block. KRN sensors have a wider initial pulse without reflection, but it was followed by extended reverberations to beyond 48 µs (Figure 6b). The peak values of the sensor output voltage are 1.6–1.84 V (measured using the input impedance of 100 MΩ). Since the peak value of V104 displacement is 11.4 nm, the peak displacement sensitivity is

(1.6~1.84)/11.4 = 0.14~0.16 V/nm = −17~−16 dB in ref. to 0 dB at 1 V/nm.

This value is about one-tenth of h_33_ coefficient of PZT-5A. This reduction is expected from the conical shape of the sensing element.

The conical FFT spectra minus the reference spectrum, shown in Figure 6c, have broad distributions, reaching the maximum at −15 dB (home-made) and −10 to −11 dB for KRN (in reference to 0 dB at 1 V/nm). These are in good agreement with the value of −14 dB that Proctor reported for his conical sensors [42]. The higher sensitivity of KRN sensors below 1 MHz is apparently due to the reverberations of the smaller backing medium. The values observed are also comparable to Greenspan’s calculation [44] of the sensitivity for a NIST conical sensor; that is, the peak of −9 dB. Considering their small size of sensing area (~1 mm in diameter), the sensitivity of the conical sensors is comparably higher than most common AE sensors. The receiving response is proportional to the area of contact (assuming the plane wave arrival) and most AE sensors have 10–100 times the sensing area of the conical elements, yet their sensitivity is only 5 to 30 dB better. 

For a similarly designed conical sensor, McLaskey and Glaser [45] reported a much higher sensitivity of 0 dB or 1 V/nm and a very flat spectrum, but their sensitivity data appear to be overly generous. The sensitivity level is at least 10 dB too high compared to Greenspan’s theoretical analysis or Proctor’s experiment of NIST conical sensors [42,44]. The reference spectrum they used was a theoretical one without validation and their spectral results require critical examination.

**Figure 7:**
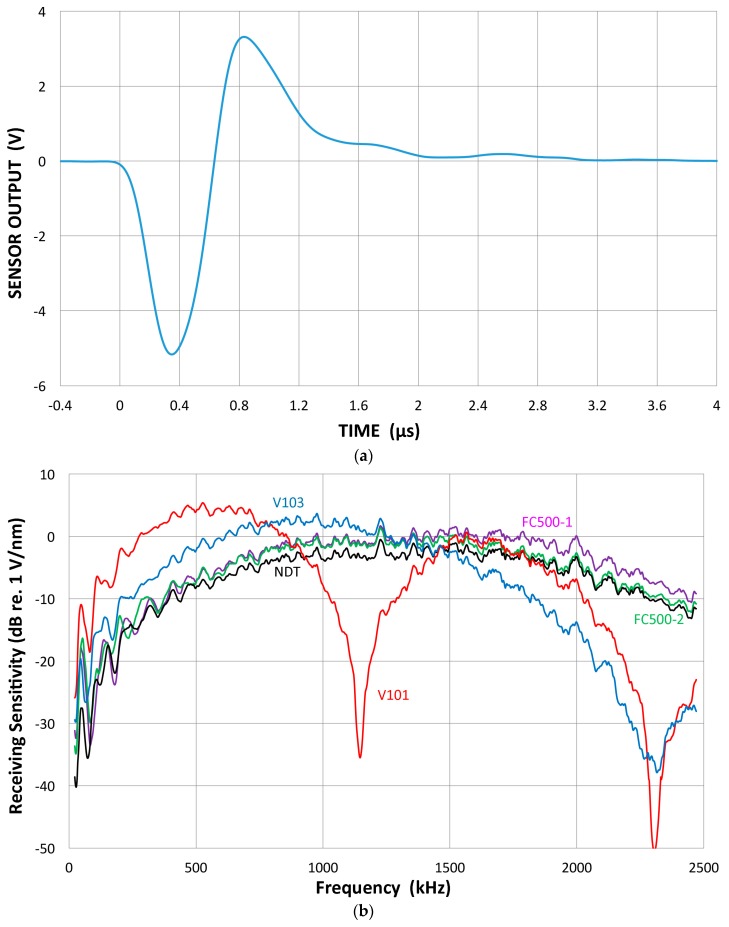
(**a**) An example of a face-to-face experiment. Received waveform of V103 from the V104 reference transmitter. Input HV pulse spectrum is given in Figure 5, curve 1; (**b**) The receiving sensitivities of five UT transducers; Olympus V101, V103, NDT Systems C16 and two of AET FC500.

Again, the direct method was used and the receiving response of five UT transducers was examined. All are well damped: Olympus V101, V103, NDT Systems C16 and two of AET FC500. From the reference (V104) into V103 receiver, we obtain the waveform shown in Figure 7a. Since V104 produces a peak displacement of 11.4 nm, the peak sensitivity of V103 is 0.453 V/nm or −6.9 dB in reference to 0 dB at 1 V/nm. The transmission-reception measurements produced spectral curves similar to Figure 2 and again the reference (V104) spectrum was subtracted. Their resultant receiving sensitivity curves are shown in Figure 7b. Three 2.25 MHz transducers show similar sensitivities as indicated by green, purple and black (C16) curves (with level shifting). These all show some sensitivities even below 100 kHz, albeit with strong variations, have a response to 2.2 MHz or more, and the peak of −2 to +5 dB sensitivity without sharp dips (except V101 near 1.1 MHz). Two lower-frequency transducers become unusable above 2.2 MHz from excessive noise. For V103, the peak is near 900 kHz at 2 dB. Thus, this value is almost 9 dB higher than the value calculated using the peak output voltage and peak displacement. The latter ignores the reflected part of the waveform, underestimating the output level by a factor of two or more.

### 3.2. Indirect Method

**Figure 8:**
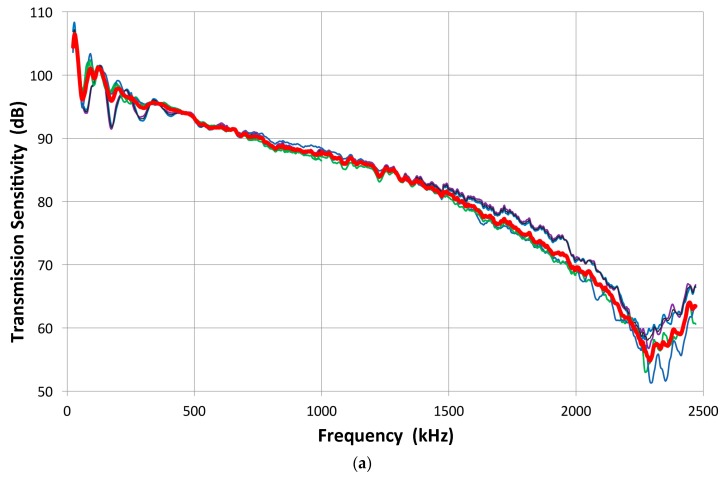

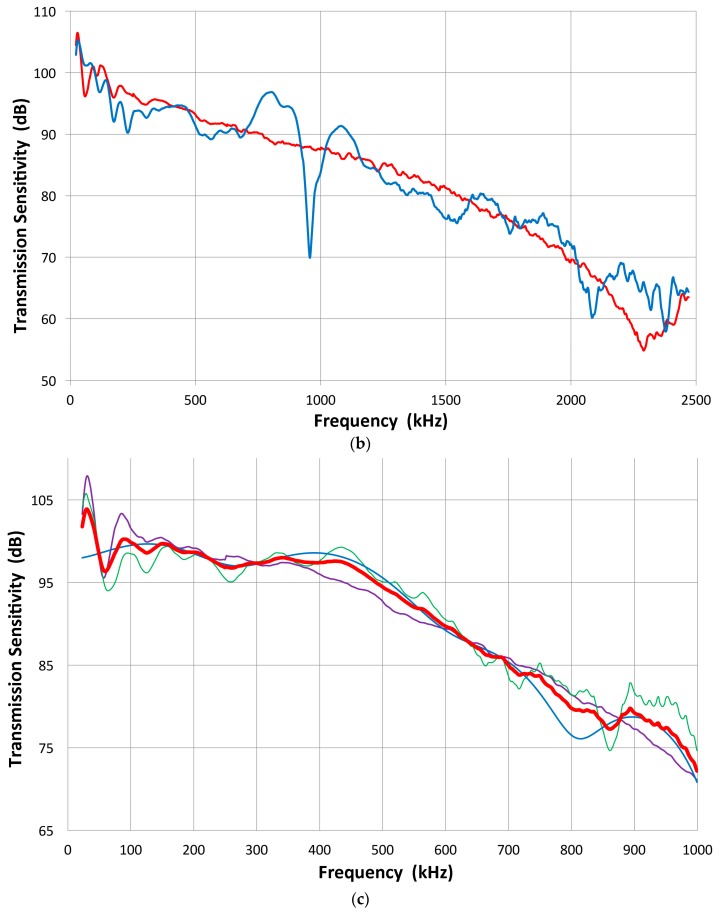
(**a**) An example of the transmitting sensitivity: for V103. The averaged spectrum is shown in a thicker red curve, while five individual curves are shown in thin curves; (**b**) Averaged transmitting spectrum of V103 (smooth red curve) compared to laser-based transmitting spectrum (blue curve, same as Figure 3b, curve 2); (**c**) Averaged receiver-based transmitting spectrum of V101 (red) and individual curves.

Next, these five transducers are used as transmitters and coupled to broadband receivers that include Olympus V104, as well as V189 (0.5 MHz), V192 (1 MHz) and V195 (2.25 MHz), all three with a 38 mm diameter. By using the combined transmitting and receiving sensitivity spectra thus obtained from set-up 2, the transmitting and receiving sensitivity spectra of any transducer can be determined. This is the “Indirect” method. In order to get the transmitting sensitivity of transducer A, couple it to transducers B, C, D, etc., with the known receiving sensitivity spectra. Since results vary slightly, these are averaged to finalize the transmitting sensitivity of transducer A. Even though the laser-based transmitting sensitivities showed some peaks and dips, the averaged spectra are generally smooth, indicating the front-face-loaded transmitting sensitivities have smooth spectra. An example of the transmitting sensitivity of V103 thus obtained is shown in Figure 8a. The averaged spectrum is shown in red while five spectra that used receiving spectra are plotted in thin curves. Except at low- and high-frequency ranges, obtained spectra converged well. In Figure 8b, the averaged spectrum is compared to that due to laser interferometry (set-up 1). These two curves mostly fit within ±5 dB except at 700–1100 kHz and above 2.1 MHz. The absence of peaks and a dip near 1 MHz indicates that the front-face loading removed extraneous vibration through the coupling of a receiving sensor. In the present case, both front-face materials are alumina plates with matching acoustic impedance. This is one of the advantages of the face-to-face arrangement (set-up 2). Another example of the indirect method is shown in Figure 8c. This is for the receiver-based transmitting sensitivity of V101. Here, three spectra were averaged to give the red curve.

**Figure 9:**
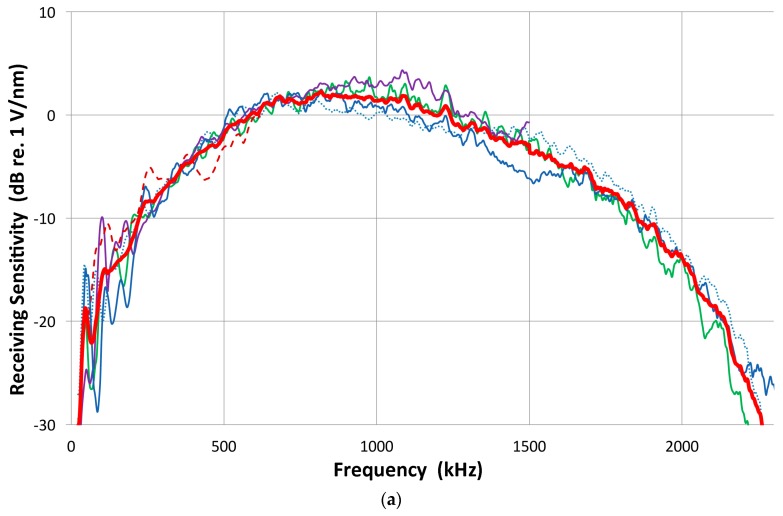

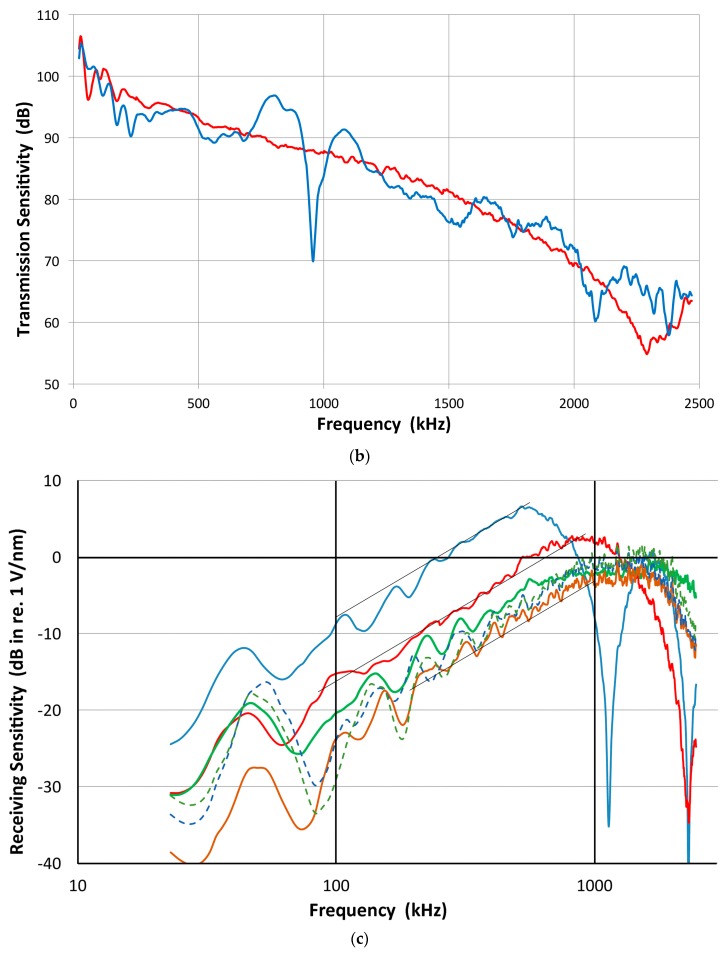

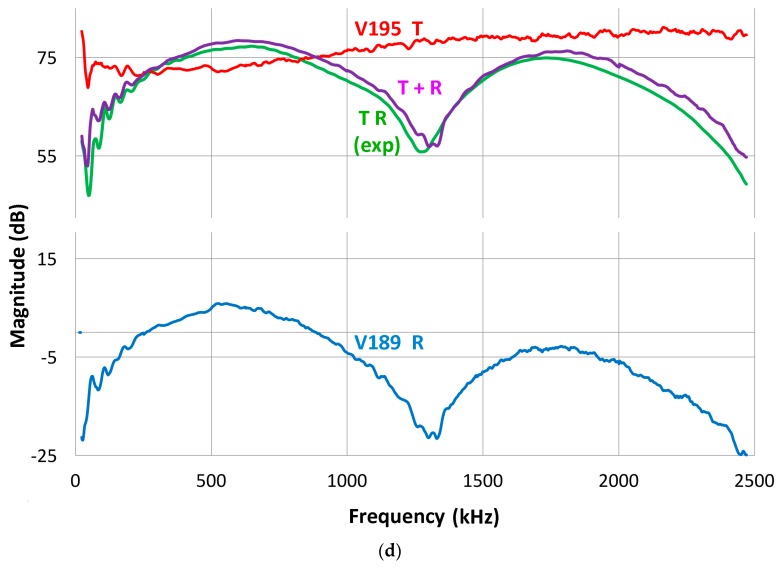
(**a**) Receiving sensitivity spectrum of V103 transducer (red), determined by averaging ones based on transmitting spectra of other transducers; (**b**) Receiving sensitivity spectrum of V104 transducer (red), determined by averaging ones based on transmitting spectra of other transducers; (**c**) Receiving sensitivity spectra of broadband transducers plotted against frequency or log *f*. These are from the direct method, except that of V104 (green). Some parts show linear *f*-dependence or flat velocity response: 70–500 kHz for V101 (blue), 80–800 kHz for V103 (red) and V104 and 0.2–1 MHz for NDT-C16 (brown); (**d**) Example of mutually consistent transmitting (V195 T: red) and receiving (V189 R: blue) sensitivities. Output of face-to-face experiment (T R (exp): green) compared to the sum of T + R (in purple).

The indirect method can be applied in reverse to determine the receiving sensitivity spectra of a transducer. For a receiver, five to six combined transmitting and receiving sensitivity spectra are determined. By subtracting the corresponding transmitting sensitivity, multiple receiving sensitivity spectra were determined and averaged. Two such examples for V103 and V104 are shown in Figure 9a,b, where thick red curves indicate the averaged spectra, while other individual spectra are plotted in thin lines. These two transducers (V103 and V104) have the peak sensitivity near 1 V/nm (within ±2 dB). This procedure is needed for V104 receiving sensitivity, but generally this is a back-up method for receiving sensitivity determination as it is further removed from the original laser interferometric calibration and errors could accumulate. Results of the two methods, direct and indirect, generally agree within 2 dB. When the receiving sensitivity spectra of broadband transducers are plotted against log *f*, as shown in Figure 9c, the spectra follow linear *f*-dependence in some ranges below the peak sensitivity. This indicates flatness in the velocity response. The extent of this behavior is limited, however: 70–500 kHz for V101, 80–800 kHz for V103 and V104, and 0.2–1 MHz for NDT-C16, as marked by thin lines. In commonly used AE sensors, this trend was absent, however. Their spectra are dominated by multiple resonance behavior.

Finding both the transmitting and receiving sensitivity spectra of a transducer gives a means of verifying the calibration. The combined transmitting and receiving sensitivity spectrum can be constructed for any combination of transducers and compared with the experiment. An example is given in Figure 9d. In this case, V189 and V195 were paired. Both are yet to get laser interferometric calibration. The V195 transmission spectrum was obtained from other transducers’ receiving spectra and is marked V195 T (red). The V189 receiving spectrum (V189 R) is the bottom curve. The sum of these two is plotted as “T + R” in purple and is compared to the output of face-to-face experiment, marked “T R (exp)” in green curve. These two agree well over 150–1200 kHz and 1.3–1.7 MHz, but have poorer agreement near the dip of the V189 receiver and above 1.7 MHz. For 12 cases examined in detail, spectral comparison yielded better results than the example in Figure 9d. The average discrepancy was typically about 1 dB except below 200 kHz or above 2 MHz, where the discrepancy is slightly higher. For validation purposes, we can also utilize laser-based transmitting sensitivity (but avoiding the frequency range that shows irregular peaks and dips: >250 kHz for V101, >650 kHz for V103, >1400 kHz for NDT C16). These were set aside in preference for using V104, but they do provide a back-up. Here, matching was moderate.

In the above comparative procedure of the combined transmitting and receiving sensitivity versus the directly measured sensitivity, it is necessary to account for the area of a receiving sensor when it is larger than the transmitter it is paired with. Assuming that the receiving sensitivity is uniform over the entire area, one adds 4.99, 7.04 and 12.04 dB for the diameter ratio of 1.333, 1.5 and 2, respectively. The present results of good matching of the experimental, combined spectrum and the one deduced from the calibration of transmitting and receiving sensitivities indicate the assumption is valid. We can thus determine mutually consistent transmitting and receiving sensitivities.

**Figure 10:**
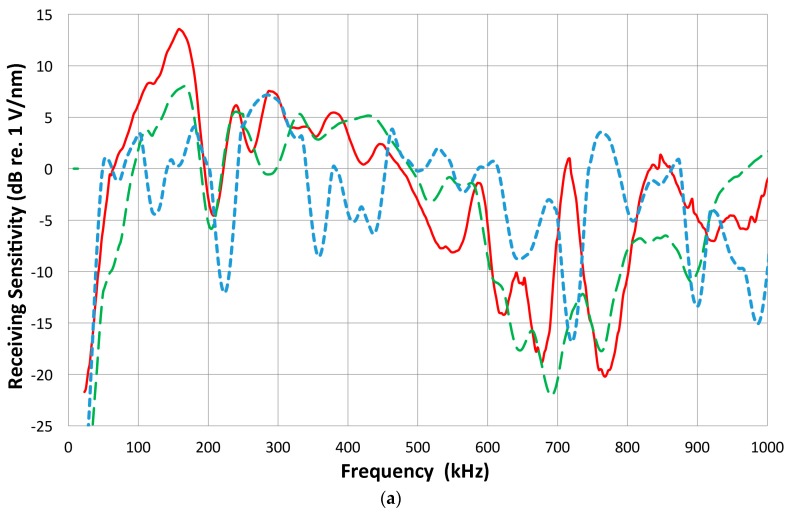

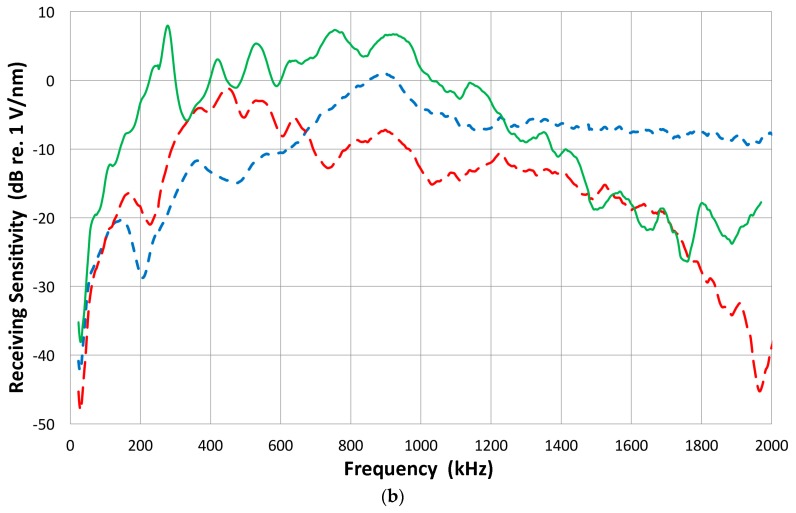
(**a**) Receiving sensitivity spectra of general-use AE sensors. PAC R15a (red), PAC R15 (green, broken), PAC R6a (blue, dash); (**b**) Receiving sensitivity spectra of general use AE sensors. PAC WD (green), PAC Pico (red, broken), PAC S9220 (blue, dash).

The procedure we used in getting the receiving sensitivity spectra of broadband UT transducers is also applicable for the calibration of general-use AE sensors. These are typically of higher sensitivity and most have more than a single resonance. Six example receiving spectra are shown in Figure 10a,b. The most sensitive of this group is PAC R15a (Figure 10a), showing a peak displacement sensitivity of 13 dB at 162 kHz. PAC R15 shows a similar spectral shape, but shows a slightly lower sensitivity. PAC R6a is a newer sensor, but this is designed for low frequency (60 kHz) surface-wave detection: here the peak is near 300 kHz. Another group (Figure 10b) shows a wideband sensor (PAC WD) with a series of resonances (270–920 kHz) at 8 dB maximum, miniature types of Pico (460 kHz peak at −1 dB, PAC) and S9220 (890 kHz peak at +1 dB, PAC). All of these general-use AE sensors have a peak sensitivity of 0 to 13 dB or about 1 to 4.5 V/nm.

Reports of displacement sensitivity of common AE sensors are scarce. McLaskey and Glaser [45] did report the peak value of PAC R15 to be 8 dB, which is in agreement with our result in Figure 10a. They used theoretical displacement at the sensor position, so the spectral data’s reliability needs to be confirmed, however.

The procedure used in our laser-based direct method is similar to that used in ASTM Standards E1106 [28]. In E1106, the receiving sensitivity is defined in terms of free displacement imposed on the sensor front face and the output voltage from the sensor. In our direct method, free displacement of the reference transducer (V104) comes from laser interferometry and this transducer is placed in contact with a sensor to be calibrated using a Vaseline couplant. The output from the sensor combines transmitting and receiving sensitivity spectra, and is given as the product of the two sensitivities. Using the decibel scale, it is the sum of the two. The receiving sensitivity can be determined by subtracting the V104 transmitting spectrum from the output. While the NIST standard displacement was measured using a capacitive sensor, we measure the reference displacement by laser interferometry. 

Our indirect method that relies on the transmitting and receiving spectra differs in principle from the NIST-based E1106. Moffatt [7] first discussed this point. Here, the displacement in the face-to-face arrangement is utilized; that is, the transmitted displacement comes from the reference transducer with the loaded front face. As shown above, these two methods produced essentially identical calibrations. By avoiding the use of those transmitters giving extraneous oscillations when free, the two methods can be used without further correction. The suppression of unwanted vibration is another benefit of face-to-face arrangement. This is enhanced by the fact that the front-face materials are usually of similar acoustic impedance.

In principle, the same calibration method is applicable for higher-frequency UT transducers. We tried laser calibration of six 5 or 10 MHz UT transducers. Perhaps because of their age (30–40 years old except the one new, V112), low high-frequency content of the HV pulse or extraneous vibration without front-face loading, the displacement output was low and combined spectra were inconsistent. The receiving sensitivities obtained showed excessive variation in shape and level (above 2 MHz). No reportable results were obtained. The method needs to be retried using newer transducers in conjunction with a faster pulser and with front-face loading with a clear medium, such as a glass cube.

### 3.3. Sensitivity Characteristics

At this stage, it is evident that the transmitting and receiving sensitivity spectra for a given transducer are different. By comparing the data for V104 (Figure 4 and Figure 9b) and for V103 (Figure 8a and Figure 9a), differences in the spectral shapes and intensity levels are clearly exhibited. In all other transducers tested, this conclusion also holds.

**Figure 11:**
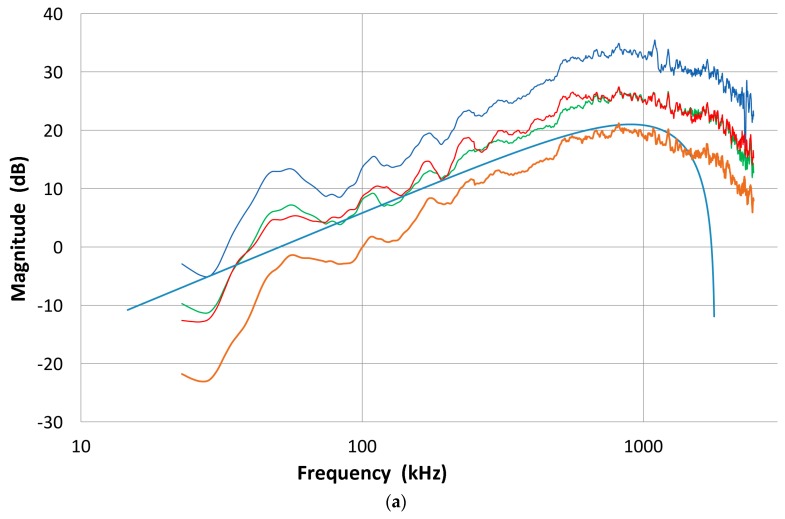

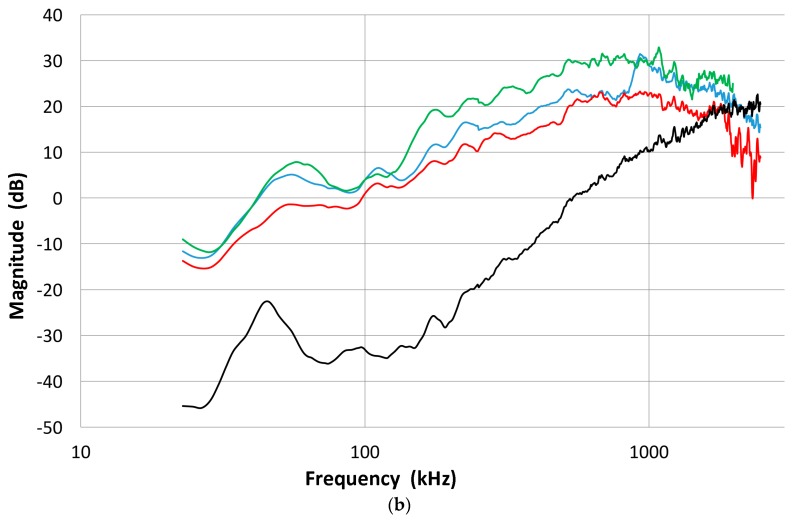
(**a**) The ratio (difference in terms of the dB scale) of the receiving sensitivity (*R*_i_) and transmitting sensitivity corrected for the HV pulse spectrum (*t*_i_) for V101 (blue curve), V103 (green), V104 (red) and NDT-C16 (brown). Smooth blue curve is the spectral ratio for single-cycle half-sine and full-sine signals; (**b**) The spectral ratio (*R*_i_/*t*_i_) for the average of four R15 and R15a (green), S9220 (blue), Pico (red) and V195 (black).

The differences between the receiving sensitivity and transmitting sensitivity (excluding the HV pulse spectrum) were calculated for 17 transducers including both broadband and resonance types. Surprisingly, the general spectral shapes of the difference (receiving minus HV-corrected transmitting spectra in decibel scale; that is, the ratio of receiving and transmitting sensitivities) were similar except for the shift in values. In terms of the symbols defined earlier for Equations (1) and (2), the magnitude plotted against the frequency represents that of R_i_/t_i_. Four examples are shown in Figure 11a for V101 (blue curve), V103 (green), V104 (red) and NDT-C16 (brown). Below about 600 kHz, the spectral difference exhibits a power law dependence with frequency; to be exact, the dependence is *f*^1.8^ for V101, and *f*^1.33^ for the rest. All show a peak around 800 kHz and start decreasing at higher frequencies. It is also strange that the middle two curves for V103 and V104 are essentially identical despite their non-matching sensitivity spectra (cf. Figure 4, Figure 8 and Figure 9). All other spectral difference curves (excluding Olympus V195) are between the curves for V101 (top) and NDT-C16 (bottom). Figure 11b shows four more plots of *R*_i_/*t*_i_–magnitude: the average of four R15 and R15a (green), S9220 (blue), Pico (red) and V195 (black). The top three curves fit within the limits given in Figure 11a, but the curve for V195 is completely different because its receiving sensitivity decreases more sharply than all others. Above 100 kHz, its frequency dependence is *f*^2.4^. Obviously, the internal design must be different from all others.

The ratio of the receiving and transmitting sensitivities of a transducer compiled here corresponds to the reciprocity parameter defined in the reciprocity calibration [15]. The reciprocity parameter is normally treated as a geometrically defined factor, independent of the transducer design, so the present observation is outside the law of reciprocity. The good match of the ratios is a required condition for satisfying the reversibility condition, stated as Equation (2). This pair of V103 and V104 indeed meets the condition, if the output received by V104 is multiplied by four (or 12 dB are added) to compensate for the diameter ratio of two. From 20 kHz to 2.5 MHz, the average difference is 1.1 dB.

The observation shown in Figure 11 can be related to the spectral difference between a half-sine monopolar displacement pulse and a full-cycle sinewave pulse (approximating a Gaussian pulse and its derivative). The difference of their FFT spectra is also plotted in Figure 11a as a smooth blue curve. The lower frequency part is linear with the frequency until it approaches the peak at 900 kHz. As we will consider later, the transmission pulse shapes from damped transducers are usually monopolar, just like a half-cycle sinewave (see Figure 3a). In contrast, the received signals tend to have an oscillatory, bipolar shape (e.g., Figure 7a). The differing transmission and reception behaviors originate from the mechanisms of pulse generation of a piezoelectric element [39] and produce the observed spectral ratio.

Here, it should be noted that the sensor output is usually terminated with 10 kΩ, which simulates typical AE preamplifiers. The cables used and the input capacitance of the digital scope (PicoScope 3405A) contribute 80 pF. In measurements involving piezoelectric elements, the impedance was at 100 MΩ with 2 pF. By using a 100× probe, the output voltage was 1–1.5 dB higher than with the 10 kΩ input impedance. In the pulse-echo ultrasonic instrument, on the other hand, this termination resistor is much smaller (5 to 50 Ω) and depends on the damping of the driving pulse generator. At high termination resistor values, we are measuring the voltage response of the sensor, while a low resistor value makes the sensor response the same as that of a short circuit or current response. We limit discussion to the voltage response or high termination resistance.

This section demonstrates that (1) the direct method of calibration is successfully used with the face-to-face arrangement, providing the receiving sensitivity of various sensors/transducers; (2) the indirect method is used to determine both the transmitting sensitivity and receiving sensitivity; (3) the indirect method suppressed extraneous oscillations of transmitters that may occur in free space; (4) the calibration results of the two methods agree well; (5) the mutual consistency of the transmitting and receiving sensitivities can be verified; and (6) the receiving and transmitting sensitivities of a transducer always differ and their ratio shows similarity among various transducers.

## 4. Piezoelectric Sensing

In order to confirm the basic sensing mode of piezoelectric sensors, we determined the response of piezoelectric elements of known piezoelectric constants and compared predicted values with observed voltage outputs. Three PZT-5A elements were used. Two disks and a cylinder were used as summarized in Table 1.

**Figure 12:**
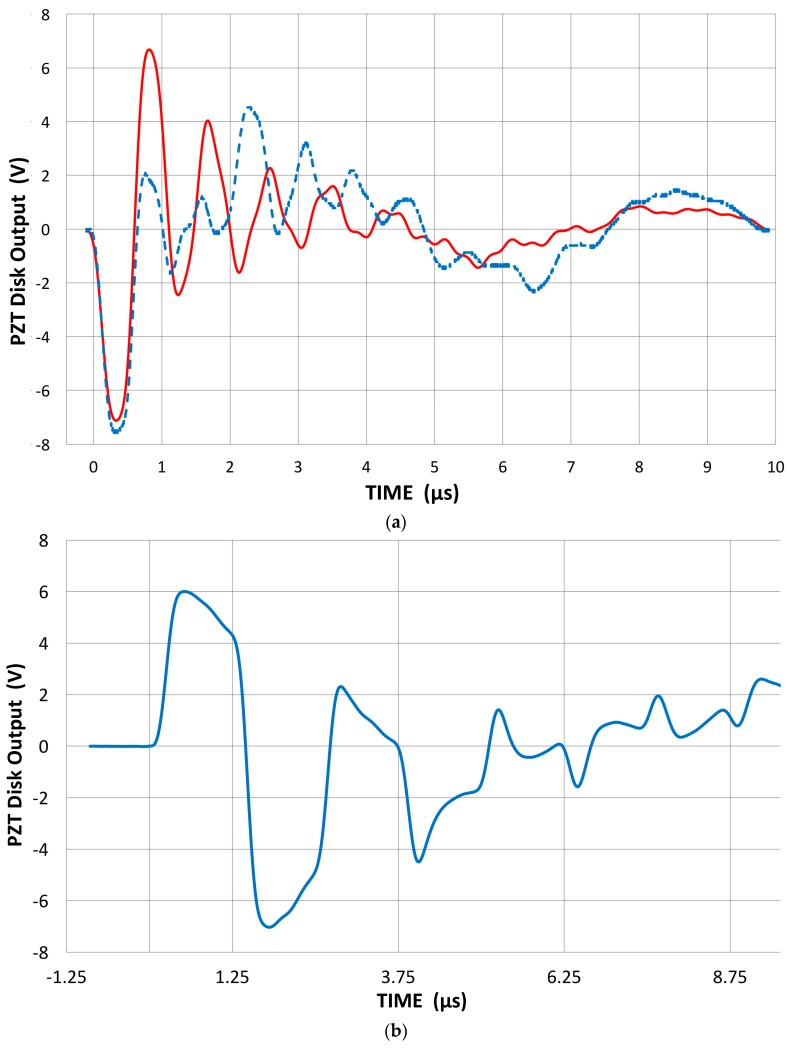

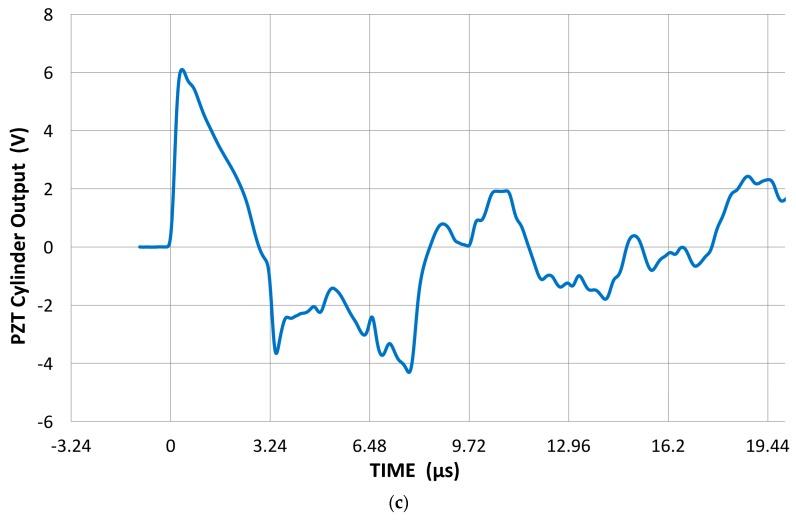
Output signals from PZT disks and cylinder, coupled to V104 reference transmitter. (**a**) Output signals from 400 kHz PZT disk; (**b**) Output signals from 160 kHz PZT cylinder; (**c**) 1 MHz disk with brass backing (blue, dash) and without backing (red).

These PZT elements were coupled on the face of V104 using a thin brass foil (60 µm thick) and Vaseline couplant. Here, three PZT output signals are shown in Figure 12, when a displacement pulse (Figure 3) with the peak value of 11.4 nm is applied. Figure 12a is for the 400 kHz disk without any backing, while Figure 12b shows the response of the cylinder that had a brass backing plate of 3.2 mm thick × 35 mm square. This backing is a minimal one, however. The disk responded with a short rise time to a peak, then the output slowly decayed, and dropped sharply upon reaching the transit time of 1.25 µs as the displacement pulse reached the back face. The elastic pulse is reflected (without backing) and the output polarity is reversed since the reflected wave now has the opposite polarity. When the initial elastic wave front reaches the back face, charges from the piezoelectric polarization are damped on the back electrode. This causes the voltage drop observed. The reflected elastic wave then carries the opposite charge to the front. At 2.5 µs, another reversal occurred. This behavior is as predicted by Redwood [39] for a PZT element without backing. The cylinder element also showed a rapid rise, but output decay was faster during the pulse transit. By the time the pulse reached the back face at 3.24 µs, the output reached a below-zero level. In this case, a brass backing plate was coupled and the effect of the reflection was halved. Again, this follows Redwood’s theory, although our backing was less than perfect and had substantial residual reflection. These two types of sensor output responses are typical of piezoelectric element behavior with or without an absorbing damper behind the back face. In fact, most transducers produce the second half-cycle (e.g., Figure 7b) even though many of them are well-damped and produce a monopolar pulse in transmission. This is apparently the cause of the nearly universal receiver-transmitter spectral ratio behavior, shown in Figure 11 and modeled by the full-cycle vs. half-cycle sinewaves. The rise times of the PZT response are 0.34–0.45 µs and 0.1 to 0.2 µs slower than the displacement pulse of V104 (cf. Figure 3) since the elastic response time has to be convolved. Thus, the input and output rise times are comparable and the output waveform before the back-face reflection is the same monopolar shape as the displacement input; it is evident that the response is not the time derivative of the input. When the reflection and subsequent reverberations are included, the response waveform can be construed as an apparent velocity-response output. Such an example can be seen in Figure 12c, for the 1 MHz disk response with and without backing, especially the latter. Again, the unbacked disk shows full-cycle oscillations, while the backed disk had a reflection amplitude of about one-third. Note that the output signals represent the 1 MHz thickness vibration of the PZT element initially, followed by radial oscillations that become dominant after 5 µs.

The observed response shows the peak output of 6.0–7.6 V (into 100 MΩ), and the observed sensitivity is 0.53–0.67 V/nm. The piezoelectric coefficient *h*_33_ for PZT-5A is 1.60 V/nm. Since the acoustic impedance mismatch from the V104 alumina face to the PZT results in a transmission coefficient of 0.766 and two couplant layers, and brass foil added an attenuation factor of 0.929, the predicted value is 1.41 V/nm. The observed sensitivity is 4.7 to 6.7 dB below the predicted response. This difference is likely from the method of piezoelectric coefficient determination that is usually conducted statically with a higher input impedance device or at much lower frequency. Thus, it is within a factor of two and the present procedure can be considered to generate voltage output in response to the displacement of a PZT element.

**Figure 13:**
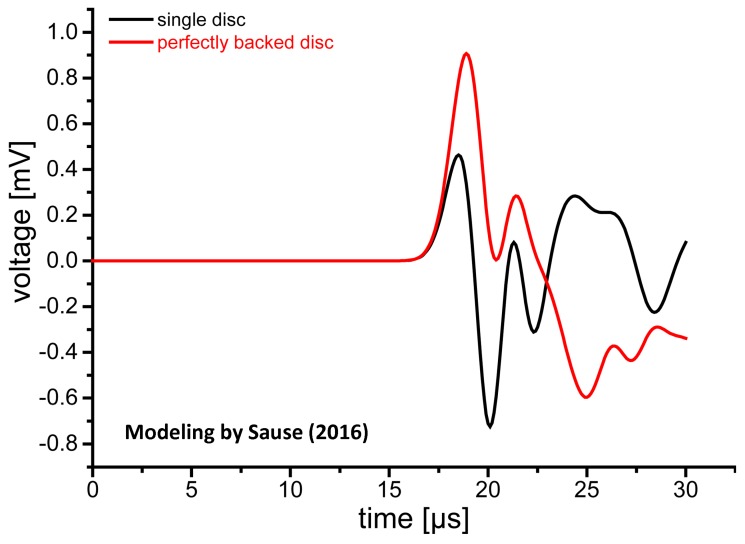
Modeling of pulse generation behavior of unbacked and perfectly backed PZT-5A disks, 3 mm thick, 10 mm in diameter. Incident plane wave is 2 µs long, cosine bell–shaped. Black: Unbacked disk case. Red: Perfectly backed disk case [46].

Recently, Sause [46] examined the pulse generation behavior of unbacked and perfectly backed PZT-5A disks (3 mm thick, 10 mm diameter) using a multi-physics modeling method [47]. He applied a cosine bell–shaped displacement of 2 µs duration. The output from the unbacked disk (Figure 13) shows a bipolar response, as expected (black curve). Note that the reflected signal is 50% higher than the initial pulse height and oscillations persist beyond 15 µs. The stronger second pulse was commonly found when the transfer functions of AE sensors were experimentally determined [38]. It was interpreted as the dumping of electrical charge at the back face from the arriving initial mechanical pulse and the reflection of an opposite polarity mechanical pulse. With perfect backing behind the disk and the PZT element terminated with 10 kΩ and 195 pF, the initial pulse is followed by an opposite polarity pulse induced by electrical impedance mismatch, as shown as the red curve in Figure 13. Because the incident elastic wave passes through the back face of the PZT element without reflection, electrical charge build-up continues until the tail end of the incident wave reaches the back face at 2.8 µs (including the propagation time through 3 mm PZT). The absence of the reflected elastic wave raises the peak value observed. The charge starts to dissipate into the termination resistor and an electrical impedance mismatch results in a bipolar output pulse. Such mismatched electrical loading conditions are the norm in the AE/UT field, and the conversion of monopolar input to bipolar output pulse shapes is prevalent. This can explain the general trend reported in the previous section, especially in connection to Figure 11. Another possible source of the bipolar response is the excitation of radial response in the PZT disk. The backing is matched for the longitudinal waves, but the radial compliances remain unmatched. More physics-based analysis of piezoelectric sensing needs to be conducted.

This part demonstrated:

(1) The origin of signal generation from a piezoelectric sensor element is best correlated to its displacement which can be measured directly.
(2) The source of bipolar signal generation is from the reflection on the back face when undamped, while for well-damped sensors it is likely to originate from the electrical impedance mismatch and partly from electrical charge transfer during elastic wave motion.

## 5. Velocity Response of a Transducer

We have shown that piezoelectric sensors generate output voltages responding to displacement input. It can still be described in terms of the time derivative of the displacement input, namely the particle velocity. The standard approach started with ASTM E1106, which treats a sensor output as proportional to the displacement function with a typical unit of V/nm. However, its FFT magnitude can be converted to express the sensitivity in reference to the input velocity function. This is accomplished by the multiplication of the 2 π*f* factor to the input function according to an identity in Fourier transform theory. When one divides the sensor output function by the velocity input function, the velocity sensitivity spectrum is obtained with a typical unit of V/m/s or Vs/m. The transmitting displacement sensitivity spectra presented earlier in Figure 3, Figure 4 and Figure 5 can be treated in this manner. In Figure 5, curve 2 is the displacement function (in nm unit), while curve 3 is the velocity function in the unit of m/s. Along with the 2 π*f* factor, remove a factor of 10^−9^ (changing from nm to m), corresponding to 180 dB subtraction on the dB scale. A note of caution: one cannot multiply 2 π*f* to a receiving displacement sensitivity spectrum in an attempt to get the velocity response. Instead, you need an opposite operation: divide by 2 π*f* and add a factor of 10^+9^.

In the AE field, it is common to find the use of µbar in place of m/s as the unit of velocity. This originated from Dunegan’s use in 1968 of a hydrophone calibration scheme in characterizing a reference transducer [48]. At that time, he took it as the acoustic pressure in water and obtained calibration up to 400 kHz. Today, the frequency limit for miniature ultrasonic hydrophone calibration is extended to 20 MHz [9].

The physical meaning of 1 µbar reference pressure has become obscure over the years, as it has not been articulated in any AE standard documents. When an immersed AE sensor receives the pressure wave, most of the wave is reflected back into water as the acoustic impedance of the sensor facing or sensing element is usually much higher than that of water. The transmitted pressure wave generates particle velocity in the sensor facing or the sensing element. However, this pressure cannot be measured. Thus, it is impractical to use it as the basis for calibration. In a recent study, Burks and Hamstad [35] concluded that the conversion procedure of the sensor response to the V/µbar reference is illogical and arbitrary unless one measures “the transient output pressure as a function of frequency from the driving transducer”.

The most logical interpretation of the V/µbar reference is that of pressure in water, as practiced in the underwater acoustics field for hydrophone calibration [49]. In the immersion tank where a hydrophone is calibrated, the acoustic pressure field is known as a function of frequency. The acoustic pressure in water is defined as the product of the acoustic impedance of water (1.48 MPa/(m/s)) and the particle velocity. Thus, the pressure of 1 µbar (=0.1 Pa) in water corresponds to 67.6 nm/s. By placing a reference transducer at the position of known acoustic pressure, it is calibrated as a function of frequency. This can then be combined with a broadband transmitter in face-to-face arrangement, from which the transmitter output can be calibrated in reference to equivalent acoustic pressure with the unit of µbar. Subsequently, a sensor under test is substituted for the reference transducer and calibrated in terms of the V/µbar reference. With this interpretation, the commonly used reference of AE sensors, 1 V/µbar, can be related to the physically-based reference of 1 V s/m. That is, 0 dB (in reference to 1 V/µbar) is 143.4 dB in reference to 0 dB at 1 V s/m. Alternatively, xx dB in reference to 1 V s/m = xx − 143.4 dB in reference to 1 V/µbar.

**Figure 14:**
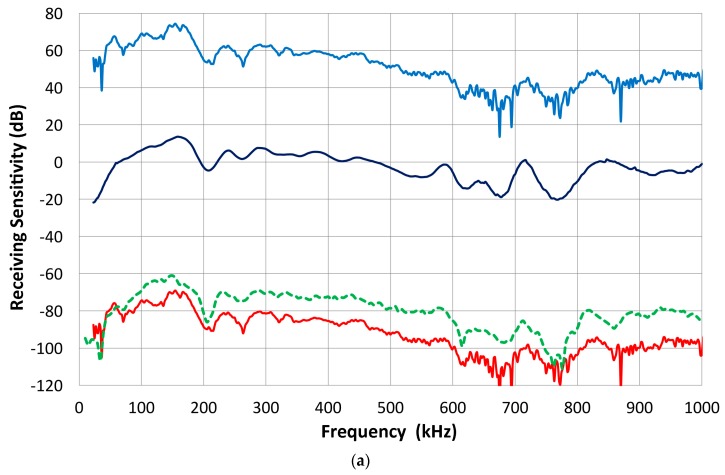

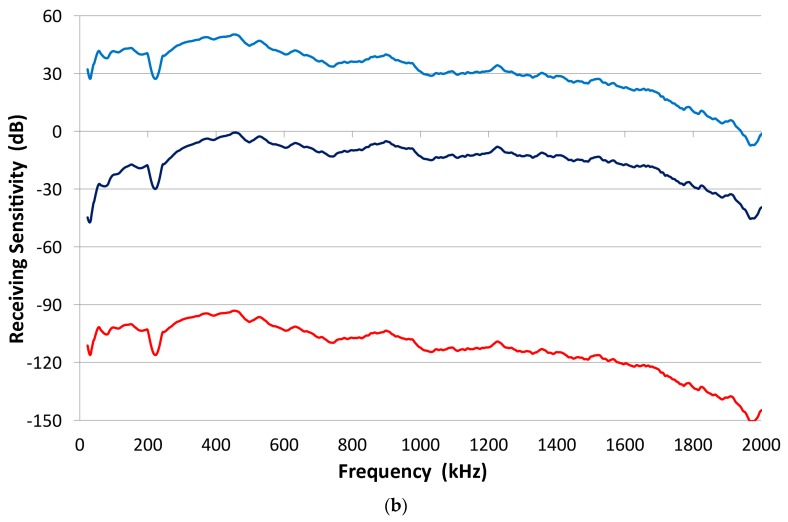
The velocity-receiving response curves for two general-use AE sensors; top (blue) in reference to 0 dB at 1 V s/m; bottom (red) after converting the reference unit to 1 V/µbar. Middle curve is the displacement-receiving sensitivity from Figure 7. (**a**) PAC R15a. Dash curve (green) is the manufacturer’s calibration curve; (**b**) PAC Pico.

Several receiving sensitivity curves for common AE sensors are shown in Figure 7. These have a peak sensitivity of around 0~15 dB in reference to 0 dB at 1 V/nm. After converting their response to volts per unit velocity of 1 m/s, we have the velocity response curves for two of them (R15a and Pico) shown in Figure 14. Curves plotted on the lower side are further converted in reference to the scale of 0 dB at 1 V/µbar. For PAC R15a, the calibration provided by the manufacturer is plotted as the green curve, just above the lower velocity spectrum. Their values are typically 10–20 dB above our calibration for >0.1 MHz. The shape of this curve matches better with the displacement calibration. This discrepancy exists for almost all other sensors examined, not just from this manufacturer, implying a possible existence of industry-wide systematic error. An interim solution is to add 72–74 dB to the manufacturer’s calibration, thereby obtaining the displacement calibration in reference to 0 dB at 1 V/nm.

**Figure 15:**
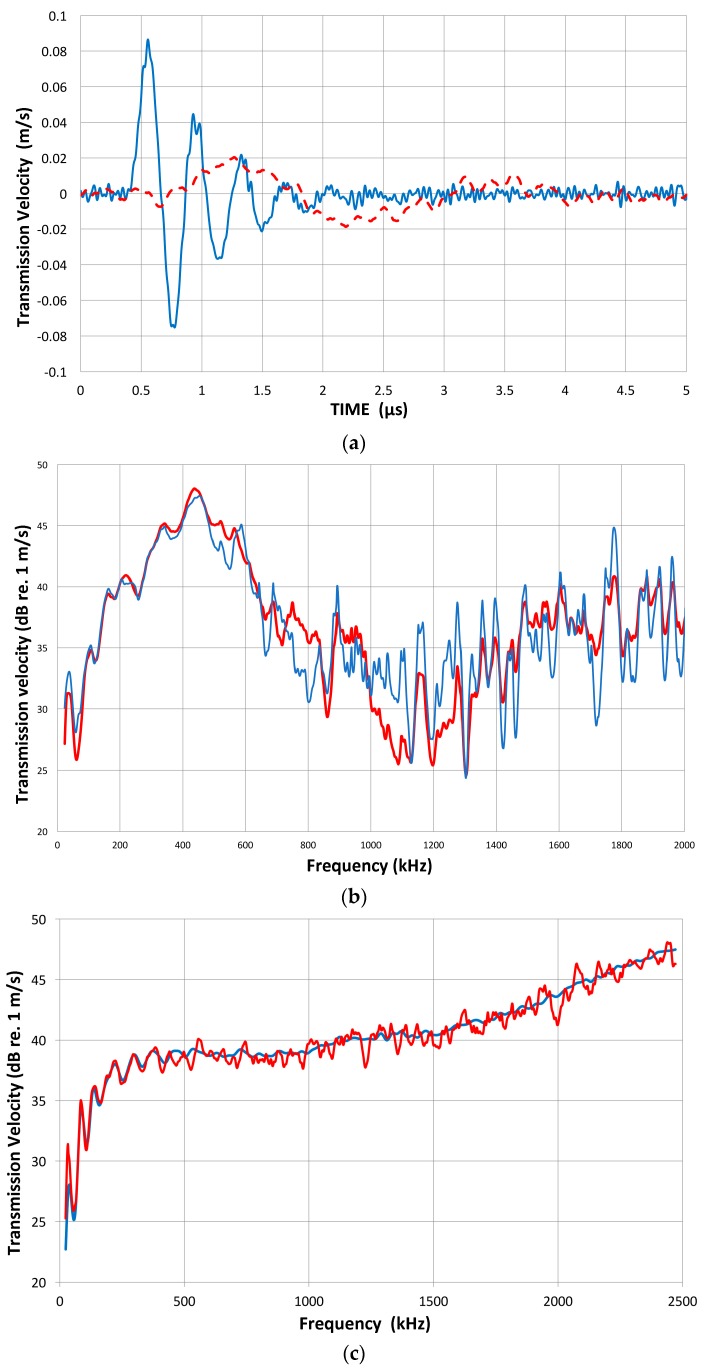
(**a**) Velocity transmission waveforms from transducers V101 (red, dash) and V104 (blue); (**b**) Velocity-transmitting spectra of V101: (blue curve). Directly differentiated spectrum, (red) 2 π*f* multiplication; (**c**) Velocity-transmitting spectra of V104: (blue curve). Directly differentiated spectrum, (red) 2 π*f* multiplication.

It is worthwhile to compare direct differentiation with the velocity conversion procedure from E1106, which we adopted here. The former gives intuitive displays in place of the abstract 2 π*f* multiplication step. A displacement waveform from a transmitter can be differentiated with time, providing a velocity waveform (or particle velocity according to the UT terminology). Two of the displacement waveforms, V101 and V104, in Figure 3a, are differentiated using the Savitzky-Golay algorithm [41] and are shown in Figure 15a (V in m/s, t in µs). The amplitude for V101 is four times smaller. The rise time (zero to peak) was reduced from 0.85 to 0.4 µs for V101 and from 0.25 to 0.15 µs for V104. The waveform changes from monopolar to bipolar. FFT peaks are at 430 kHz and at 2.6 MHz, respectively, close to their nominal values (Figure 3 and Figure 5). These peaks are more pronounced than the original displacement spectra. Other UT transducers give similar results. The FFT spectra are compared with the 2 π*f* multiplication curves in Figure 15b,c. The differentiated V101 spectrum (blue curve) is noisier, but it follows the 2 π*f* counterpart (red) up to 700 kHz (Figure 15b) well. The differentiated V104 spectrum (blue, Figure 15c) also matches the 2 π*f* counterpart (red) to 2.5 MHz and is smoother than the 2 π*f* curve. It is clear that the velocity conversion procedure from E1106 is valid and is simpler. However, the direct differentiation does provide the velocity waveform and numerical values without the inverse FFT procedure.

The above discussion shows that it is unproductive to classify a sensor to displacement response or velocity response without specifying the frequency range or the flatness of response. NIST-type conical sensors with a large-mass backing clearly have a broad range of flat displacement response, while typical accelerometers are designed to produce nearly flat wideband acceleration response below the resonance frequency. Common AE sensors have been designed for resonance-based peak sensitivity, while newer designs start to broaden the peak sensitivity ranges.

This section leads to

(1) The clarification of pressure calibration references in terms of 1 V/(m/s) and 1 V/µbar.
(2) Demonstration of the equivalence of two conversion methods to velocity.

## 6. Calibration Methods Using Three Transducers

### 6.1. Reciprocity Calibration Methods

The laser-based calibration methods have demonstrated that the transmitting and receiving sensitivities of UT and AE transducers differ. Hill and Adams [19] applied the basic reciprocity principle to the cases appropriate for contact piezoelectric transducers, starting from the classic reciprocity calibration methods. In their analysis of reciprocity calibration methods, transmitting and receiving sensitivities are not required to be identical. This exactly fits the experimental reality established above. Their analysis led to the receiving sensitivity of the No. 2 sensor, *R*_2_, to be given by the Hill-Adams equation in the face-to-face method,

*R*_2_ = [(*E*˚_12_ × *E*˚_32_/*E*˚_13_)(*R*_3_/*T*_3_)]^1/2^, (6)

and for pairs coupled through the transfer block

*R*_2_ = {[(*E*_12_ × *E*_32_)/(*X* × *E*_13_)](*R*_3_/*T*_3_)}^1/2^. (6a)

Here, *X* is the transfer function of the propagating medium.

As noted earlier, this result leads to the demise of reciprocity calibration methods for piezoelectric contact transducers since there are six unknowns and only three equations. This is a clear violation of the mathematical principle. In order to use the Hill-Adams equation, *R*_3_ and *T*_3_ must be determined by other means, such as laser interferometry. Thus, the reciprocity calibration lost their main claim of convenience: only electrical measurements are needed.

Another extension of the experimental transmission-reception curves using set-up 2 (cf. Figure 2) is to determine the longitudinal wave reciprocity parameter (defined above as *X*) for a particular pair of transducers and a transfer block. This corresponds to set-up 3 in Figure 1a. Theoretically, this has been shown to be linearly frequency-dependent. Usually, it has been considered to be in the far-field condition and diffraction corrections were often ignored. This parameter corresponds to the attenuation of the longitudinal wave as it passes the transfer block. Thus, one can obtain a face-to-face response of a transducer pair, *E*˚_ij_, as well as with the transfer block between the transducer pair, *E*_ij_; then, *X*_ij_ = *E*_ij_/*E*˚_ij_. As usual, pulse excitation was used and the transfer block was of Al 7075 alloy (300 × 300 × 156 mm^3^).

**Figure 16:**
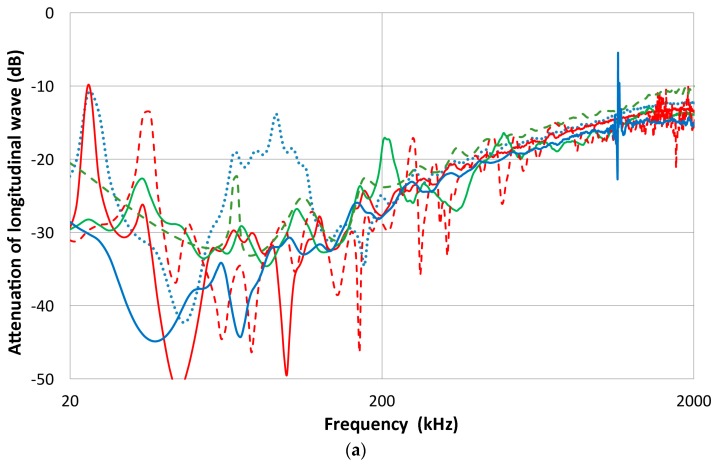

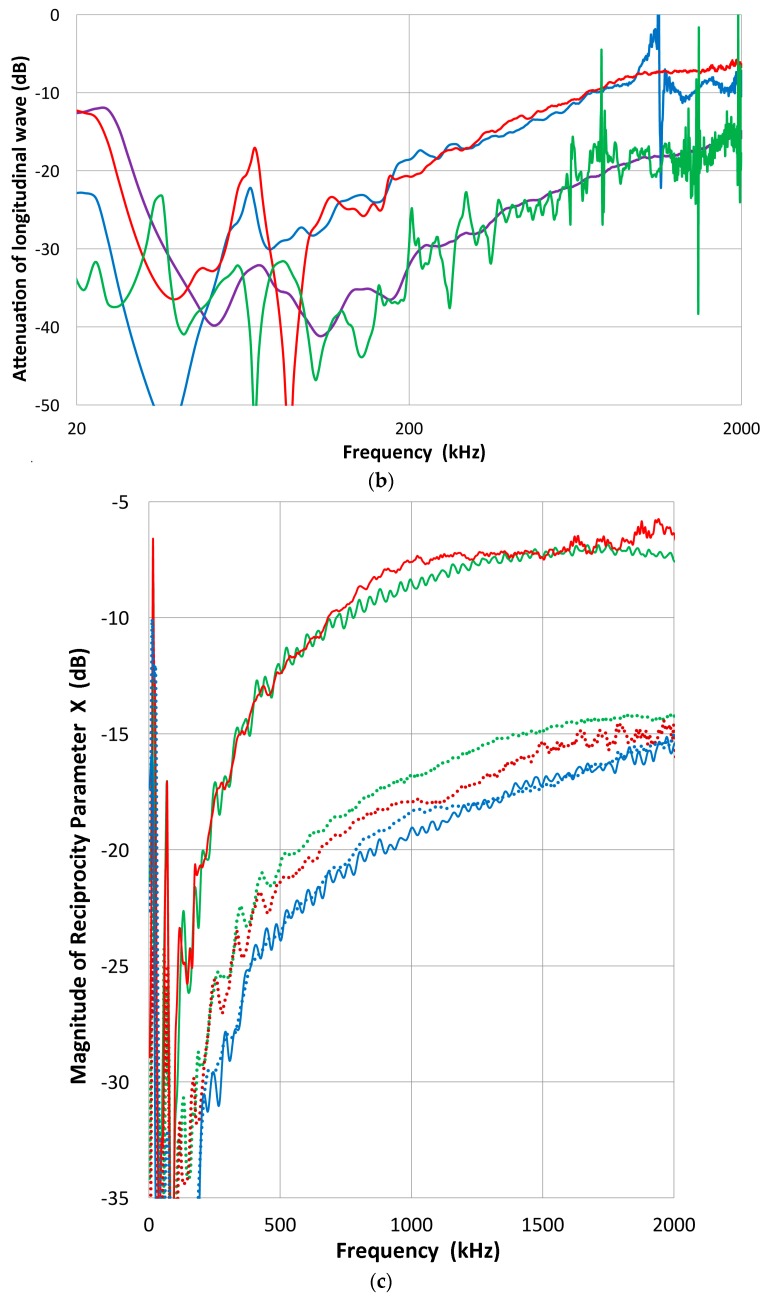
Experimental longitudinal wave reciprocity parameters X for two transmitters using the Al block; (**a**) V104 transmitter and six receivers: V101 (blue), V103 (green, dash), FC500-1 (blue, dot) and -2 (red), WD (red, dash) and S9220 (green); (**b**) V103 transmitter and four receivers: V104 (red), V101 (blue), R15a (green), NDT-C16 (green); (**c**) Reversibility of transmission-reception pairs of transducers examined using three pairs of V103-V104 (red curves), V103-NDT (blue curves) and V104 and NDT (green curves). Only the V103-NDT pair was reversible. Solid red and green curves show the cases with V104 as the receiver.

Results using two transmitters (V103 and V104) for a total of 10 combinations are shown in Figure 16a,b. The V104 transmitter produced spectral curves in Figure 16a with mostly broadband receivers (V101, V103, FC500-1 and -2, WD and S9220). Above 150 kHz, the curves tend to fall within a band with decreasing slope. Approximate frequency dependence appears to be *f*^0.7^. At lower frequencies, a general trend appears to be of flattened attenuation, indicative of diffraction effects. In addition, large fluctuations occur with strong effects from transducer sensitivities and diffraction at large wavelengths. At 200 kHz in Al, the wavelength is at ~30 mm, larger than most transducers; at 20 kHz, it is 300 mm and equals the size of the block we used. Here, all the receivers were of equal or smaller size than the transmitter (25 mm). When the V103 transmitter was used, two groups of reciprocity parameters were observed (Figure 16b). The upper group was of larger 25-mm-diameter receivers, while the lower group was of the same 13-mm size as V103. The slope above 200 kHz was similar, but again the slope decreased above 1 MHz. The theoretical reciprocity parameter is to be a single function, independent of the size of either the transmitter or receiver. Clearly, the present results are inconsistent with the theoretically obtained function for point source to point receiver geometry [16,17].

When the transmission-reception pairs of transducers are reversed, the reciprocity principle anticipates reversibility. This behavior is examined using the three pairs of V103-V104 (red curves), V103-NDT (blue curves) and V104 and NDT (green curves). Only the V103-NDT pair has identical transducer diameters. Results are shown in Figure 16c. Only the V103-NDT pair was reversible. The average difference in magnitude of *X* is 0.3 dB between 100 and 2 MHz, although large variations occur below 100 kHz. The other pairs were not reversible. The average difference in *X* was 9.2 dB for the V103-V104 pair and 7.9 dB for the V104-NDT pair, respectively. In the face-to-face calibration, we can correct for sensor size under the uniformity assumption, but this procedure did not work here. This is partly due to spherical waves arriving at the back face of the transfer block. Beam spread is also dependent on the transmitter size. This result adds another reason to avoid reciprocity calibration methods that must rely on a transfer block. Instead of a single reciprocity parameter *X*, the Hill-Adams equation must now be modified to include the ratio of [*X*_13_/(*X*_12_ × *X*_32_)]^1/2^. These can be measured as described above. With this change, the Hill-Adams equation takes the form of

*R*_2_ = {(*X*_13_ × *E*_12_ × *E*_32_)/(*X*_12_ × *X*_32_ × *E*_13_)](*R*_3_/*T*_3_)}^1/2^. (6b)

This equation can further be simplified, since *E*_ij_ = *E*˚_ij_ × *X*_ij_ from. Equations (3) and (3a). Upon substitution, Equation (6b) is reduced to Equation (6). Consequently, the use of the transfer block is not required and our evaluation of *X* parameters was unnecessary. The method using Equation (6) and set-up 2 will be considered in the next section.

**Figure 17:**
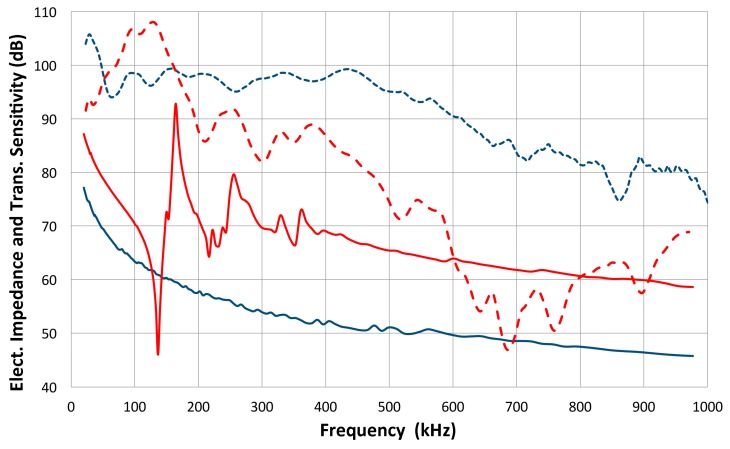
The magnitude of the complex impedance |*Z*| and transmitting sensitivity (*T*_x_) of V101 (blue) and R15 (red) transducers; |*Z*| is plotted with solid curves and *T*_x_ in broken curves. The unit of |*Z*| is Ω and shown in dB.

Another aspect of the reciprocity calibration method needing further consideration is the use of transmitter electrical impedance for representing the transmitter output. Sometimes, the current into the transmitter is used for the same. We measured the electrical impedance, *Z*, of several transducers with or without front-face loading. An Al block under ~1 kg force was coupled with Vaseline couplant for loading experiments. The frequency range was from 20 kHz to 1 MHz. Results of the magnitude of the complex impedance are shown in Figure 17 for a damped UT transducer (V101, 0.5 MHz) and a resonant AE transducer (R15, 160 kHz). These utilized the Bio-Logic VMP-3 potentiostat. The effect of front-face loading was minor and visible only on phase spectra (not shown). V101 showed a basically capacitive response, slowly decreasing |*Z*| with frequency. The R15 transducer showed sharp peaks and dips. In all six transducers tested, no correlation was found with the transmitting spectra. It is thus concluded that the use of transducer electrical impedance or input current serves no valid purpose for identifying the transmission characteristics. The transmitter output of a transducer must be measured by physical methods.

### 6.2. Tri-Transducer Method

**Figure 18:**
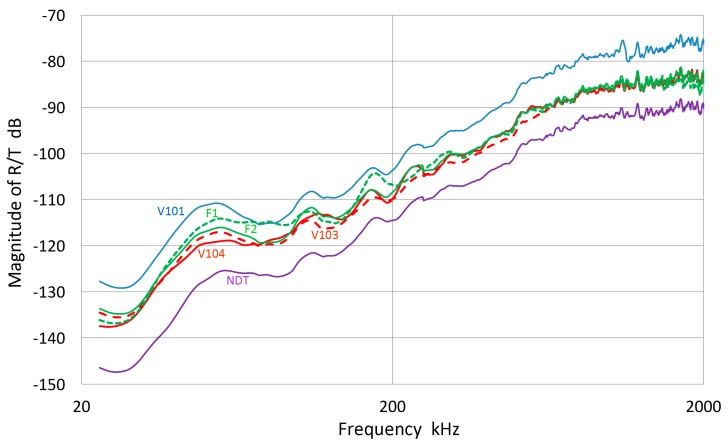
The magnitude of R/T ratios of six transducers. V101 (blue), F2 (FC500-2, green), F1 (FC500-1, green-dash), V104 (red), V103 (red, dash), NDT (purple).

Previously, the ratio *R*_3_/*t*_3_ was shown in Figure 11. This was frequency-dependent and varied from transducer to transducer, although its dependence was relatively insensitive to transducer types, but with a ±10 dB amplitude variation. The ratio *R*_3_/*T*_3_, considered here, includes the FFT spectrum of the high-voltage electric pulse. Figure 18 shows six *R*/T curves of broadband transducers. The top curve is for V101 and the bottom one is for NDT. Four others are in the middle. Most R/T curves fall between those of V101 and NDT with the exception of V195 (lower and steeper than NDT). The frequency dependence is higher and is approximately *f*^5/3^ over the range of 0.1 to 1 MHz. In the course of our laser-based calibration experiment, we have obtained *T*_i_, *R*_j_ and *R*_j_/*T*_j_ for over 25 transducers. *E*˚_ij_ data has also been accumulated for many combinations. Thus, it is worthwhile to compare the receiving sensitivity curves of a few sensors between our laser-based calibration methods and Equation (6) involving three transducers. The latter is designated as the tri-transducer calibration method (though this also needs laser assistance).

**Figure 19:**
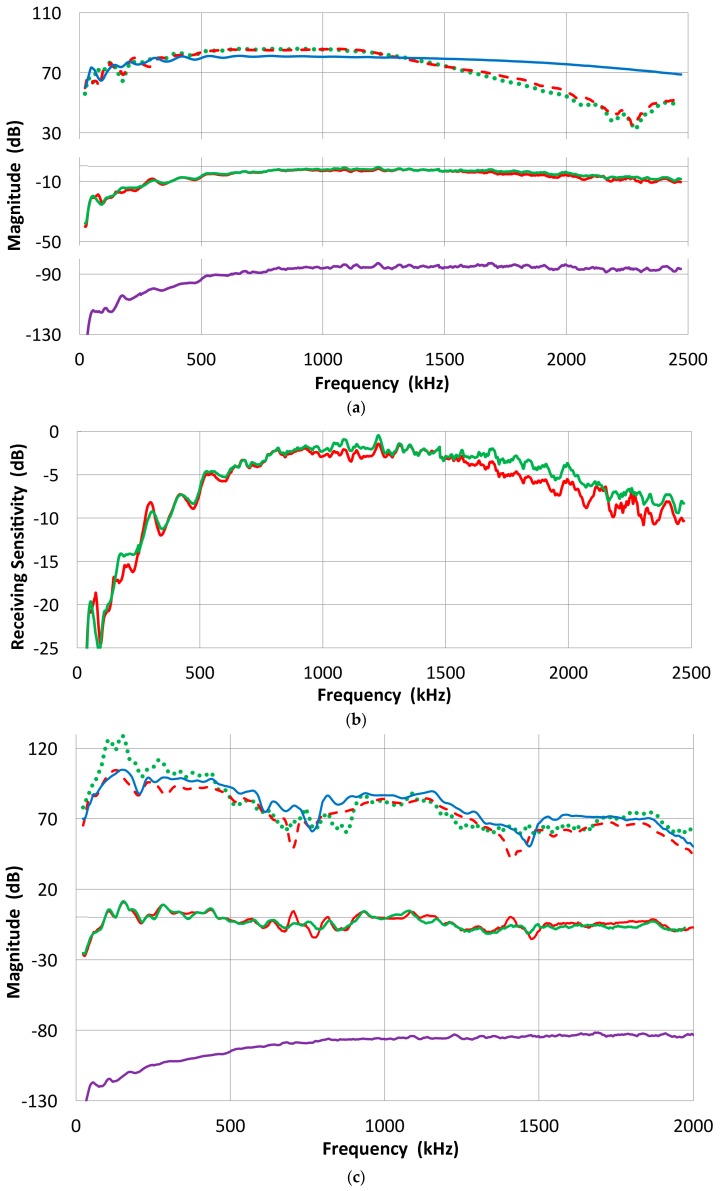

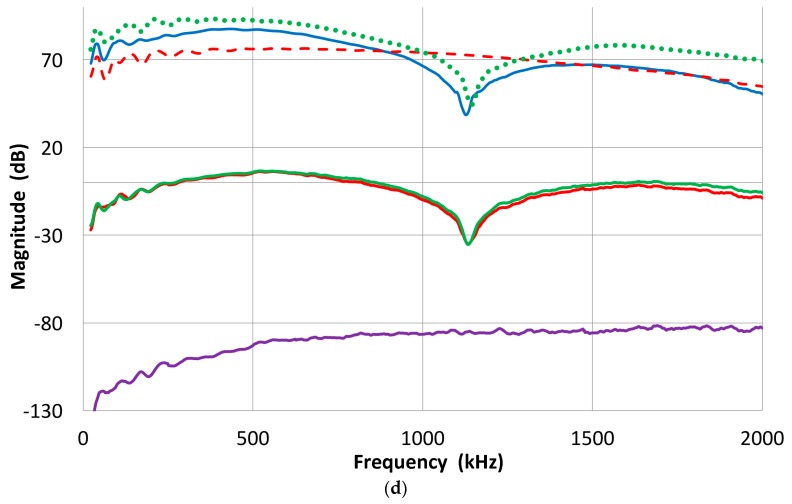
Comparison between laser-based and tri-transducer calibration methods. Style code for curves: Green dot = #1 to #2; red dash = #1 to #3; Blue = #3 to #2. tri-transducer receiving sensitivity = Red; Laser-base receiving sensitivity = Green; Ratio for #3, *R*_3_/*T*_3_ = Purple. (**a**) For transducers: (1) 1 MHz UT (SF 01); (2) FC500-2 and (3) FC500-1; (**b**) Comparison of laser-based (green) and tri-transducer (red) receiving sensitivity for FC500-2; (**c**) For transducers: (1) PAC R15; (2) PAC R15a and (3) V103; (**d**) For transducers: (1) V103; (2) V101 and (3) V104.

Figure 19a shows the case for (1) 1 MHz UT (SF 01); (2) FC500-2; and (3) FC500-1. The spectra for three *E*˚_ij_ (top group), *R*_2_ (FC500-2) and the same from the laser method (middle), and *R*_3_/*T*_3_ for FC500-1 (bottom curve) are plotted. The values of *R*_2_ of the two methods agree quite well below 1.5 MHz with the average difference of 0.34 dB, as can be seen in Figure 19b. The dip in the two *E*˚_ij_ curves is from #1, but it has no effect on the outcome. Figure 19c is for the case with (1) PAC R15; (2) PAC R15a; and (3) V103. The three *E*˚_ij_ curves are full of peaks and dips as #1 and #2 are resonant sensors. Again, the values of *R*_2_ for R15a by the two methods are close. Here, the average difference below 600 kHz was 1.00 dB and 1.5 dB to 1 MHz. Considering the many peaks and dips in the spectra, this is a good match. These two cases show that the choice of transducer 1 is non-critical: it just needs to transmit in the frequency range of interest. The third example (Figure 19d) has three UT transducers: (1) V103; (2) V101; and (3) V104. Curve designation remains the same. Here, V101 has a major dip in its receiving sensitivity at 1.1 MHz. Over 50 to 650 kHz, the average difference was 0.44 dB, while it rose to 1.0 dB to 1 MHz and 1.7 dB to 2 MHz even including the dip range. The tri-transducer method has been used for over 20 more combinations of three transducers. The average difference (over 22 kHz to 2 MHz) with the result of the direct method is typically less than 0.5 dB, although a few cases show values of 1 to 2 dB as in the two examples above.

The tri-transducer method can be used to obtain the transmitting sensitivity *T*_1_ similarly to the indirect method. We have

*T*_1_ = [(*E*˚_12_ × *E*˚_13_/*E*˚_32_) (*T*_3_/*R*_3_)]^1/2^. (7)

Using this equation and three transducers of V104, NDT and V103, the transmitting spectrum for V104 was obtained. The average difference with that of the direct method was 0.12 dB over 22 kHz to 2 MHz. With this method for getting *T*_i_, however, the use of some resonant sensors as a receiver produced poor results and should be avoided. This approach provides a new means of verifying the laser interferometry and the front-loading effects.

This tri-transducer calibration method is beneficial for its reliance on the sensitivity ratio and avoiding the direct use of the displacement transmission reference alone. Reception from another transmitter is included so we can avoid potential problems that may arise from a particular combination of transducers. Still, this approach does require the determination of the *R*_3_ /*T*_3_ ratio of a transducer by laser interferometry.

Conclusions from this section are:

(1) With demonstrated differences in the transmitting and receiving sensitivities of UT and AE transducers, the tri-transducer calibration method based on Equation (6) in combination with the physically measured ratio of *R*_3_ /*T*_3_ replaces reciprocity-based methods.
(2) The proposed approach of reciprocity calibration methods for AE sensors, only using electrical measurements, is invalid, even if the sensors are reversible. Experimentally obtained longitudinal wave reciprocity parameter *X* varies depending on the transducer pair used and not invariant as the reciprocity theory requires.
(3) The reciprocity parameter *X* and the use of a transfer block are found to be unnecessary.

## 7. Conclusions

Outstanding issues of sensitivity calibration methods for ultrasonic and acoustic emission transducers have been examined. Determining spectral sensing properties is of utmost importance, especially in AE sensors, but recent research activities in this area have been low. In addition, today’s emphasis in this field has been to model the sensor behavior using the lumped parameter approach so that it can be integrated into systems modeling. On the other hand, physics-based analysis of piezoelectric sensing has been limited until recently. With new tools available today, such as laser interferometers and advanced modeling methods, we are closer to the goal of finding a suitable and workable approach to transducer calibration and clarifying underlying sensing mechanisms.

Laser-based displacement measurement leads to the determination of transmitting sensitivities of transducers. While simple in concept, some transducers generate extraneous vibrations on the front surface when it is free from solid contact. This issue was overcome by a suitable selection of a transmitter and by using an indirect method through the use of receiving sensitivities of other transducers. It was then possible to obtain mutually consistent transmitting and receiving sensitivities. The results also establish the foundation for face-to-face calibration methods, which were beset by the uncertainty of input parameters without access to the transmitter face. Further, it is discovered that the receiving and transmitting sensitivities of over 20 transducers are always different, while their ratios exhibit unexpected similarity. The latter characteristics are traced to the monopolar pulse generation of damped piezoelectric transducers as a transmitter and, as a receiver, bipolar received signals due to the reflection on the back face. This occurs even in transducers with good backing, likely from electrical impedance mismatch and charge transfer during elastic wave motion.

The observed difference in the receiving and transmitting sensitivities of a transducer leads to the invalidation of reciprocity calibration methods for piezoelectric contact transducers. The issue was raised in 1979 [19], but users of reciprocity calibration have ignored the fact that a separate measurement of the ratio of the transmitting and receiving sensitivities is required. We have also measured the reciprocity parameters *X* in the case of through-transmission and found this to be dependent on transducer pairings, sizes, frequency, etc., in direct conflict with its definition in the reciprocity calibration methods. These are also not reversible in cases of different-sized pairs. In the end, however, parameters *X* and a transfer block were found unnecessary. In place of the reciprocity calibration, the tri-transducer method with a face-to-face set-up has emerged to be a validated calibration procedure.

Displacement vs. velocity calibration terminology is examined, in view of the ill-defined “V/µbar” reference used in the commercial reporting of sensor properties. It even found a way into standard documents [35]. Returning to the origin of its introduction by Dunegan [48], the definition from hydrophone calibration standards clarified the reference. The procedure is given for converting between the velocity sensitivities in the reference to the unit velocity of 1 m/s and to the acoustic pressure of 1 µbar in water.

It is demonstrated that three methods discussed here, the direct, indirect and tri-transducer methods, provide reliable sensor calibration results that are consistent among them.

## Figures and Tables

**Table 1:** Piezoelectric elements used.

PZT-5A Element	Nominal Frequency	Height (mm)	Dia. (mm)	Transit Time (µs)
Disk 1	1 MHz thickness mode	2.10	12.7	0.454
Disk 2	400 kHz thickness	5.35	18.3	1.25
Cylinder	160 kHz thickness	14.0	10.0	3.24
