# Correction: Ono, K. Calibration Methods of Acoustic Emission Sensors. *Materials* 2016, *9*, 508

**DOI:** 10.3390/ma9090784

**Published:** 2016-09-20

**Authors:** Kanji Ono

**Affiliations:** Department of Materials Science and Engineering, University of California, Los Angeles (UCLA), Los Angeles, CA 90095, USA; ono@ucla.edu; Tel.: +1-310-825-5534

The author wishes to make the following corrections to this paper [1].

The published Figure 9b was incorrect. The correct Figure 9b is shown below.

The order of sub-graph in Figure 12 was incorrect. The correct order: Figure 12a should be Figure 12c; Figure 12b should be Figure 12a and Figure 12c should be Figure 12b. The correct order of Figure 12 is shown below.

On page 27, line 5, “Figure 2a” should be “Figure 3a”.

On page 32, the caption for Figure 17 “V101 (red) and R15 (blue)” should be “V101 (blue) and R15 (red)”.

The authors regret any inconvenience or misunderstanding caused by these errors. The manuscript will be updated and the original will remain available on the article webpage.

## Figures and Tables

**Figure 9:**
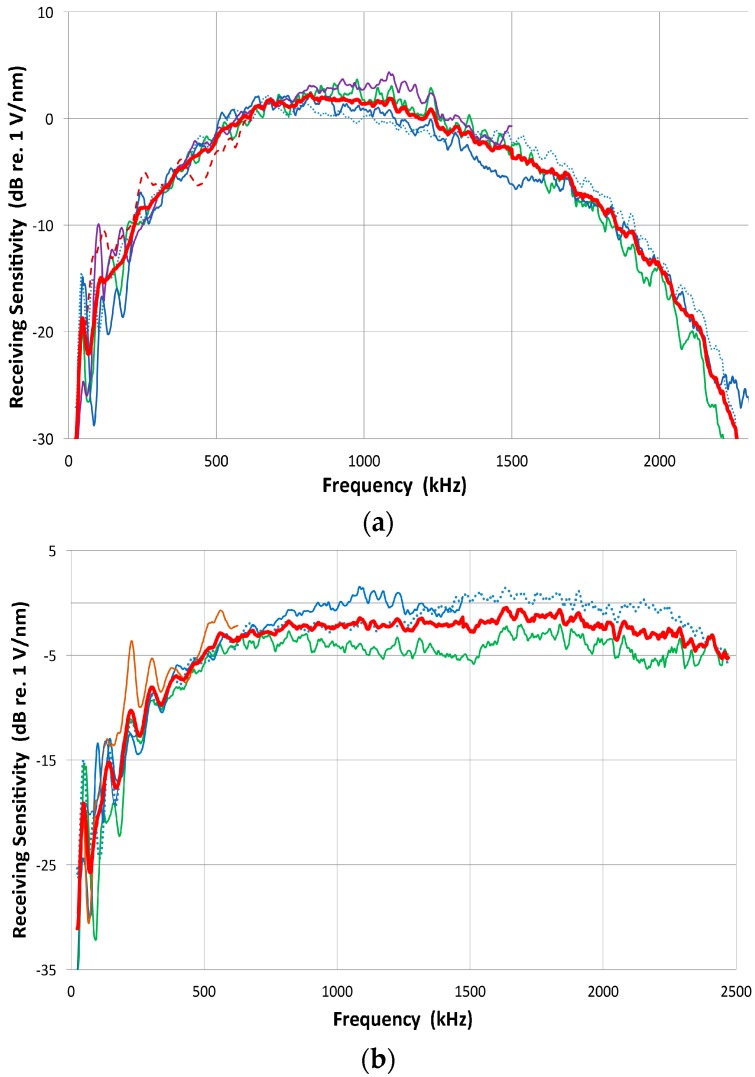

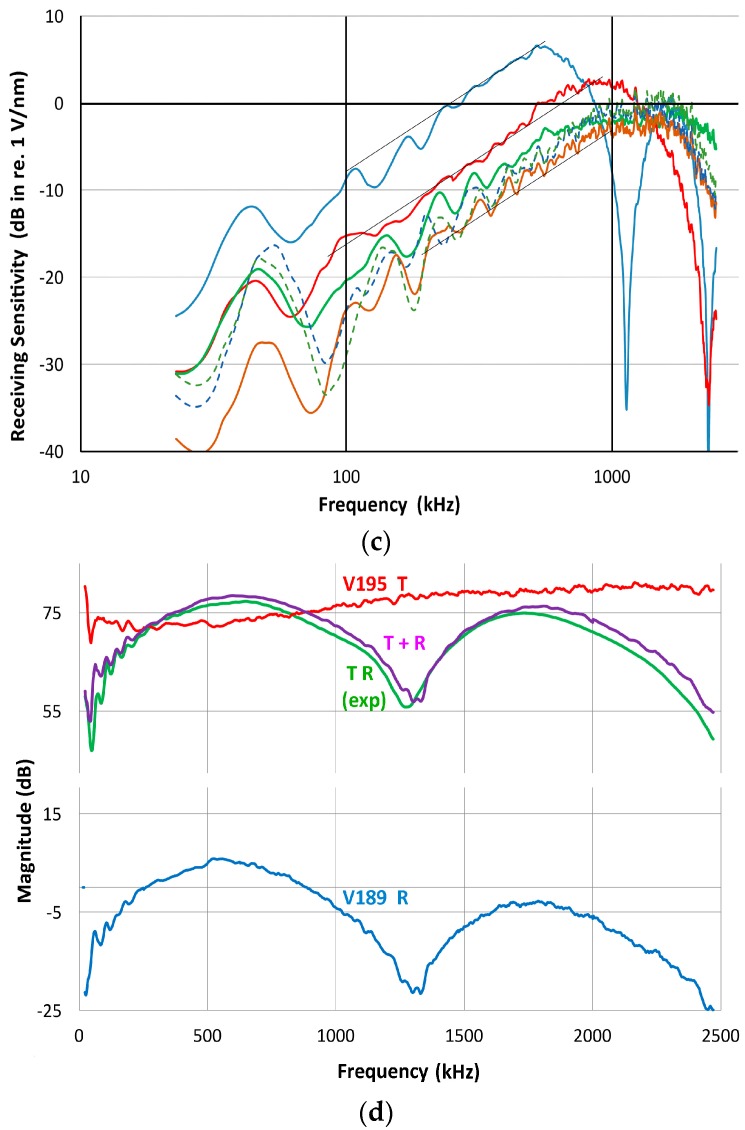
(**a**) Receiving sensitivity spectrum of V103 transducer (red), determined by averaging ones based on transmitting spectra of other transducers; (**b**) Receiving sensitivity spectrum of V104 transducer (red), determined by averaging ones based on transmitting spectra of other transducers; (**c**) Receiving sensitivity spectra of broadband transducers plotted against frequency or log *f*. These are from the direct method, except that of V104 (green). Some parts show linear *f*-dependence or flat velocity response: 70–500 kHz for V101 (blue), 80–800 kHz for V103 (red) and V104 and 0.2–1 MHz for NDT-C16 (brown); (**d**) Example of mutually consistent transmitting (V195 T: red) and receiving (V189 R: blue) sensitivities. Output of face-to-face experiment (T R (exp): green) compared to the sum of T + R (in purple).

**Figure 12:**
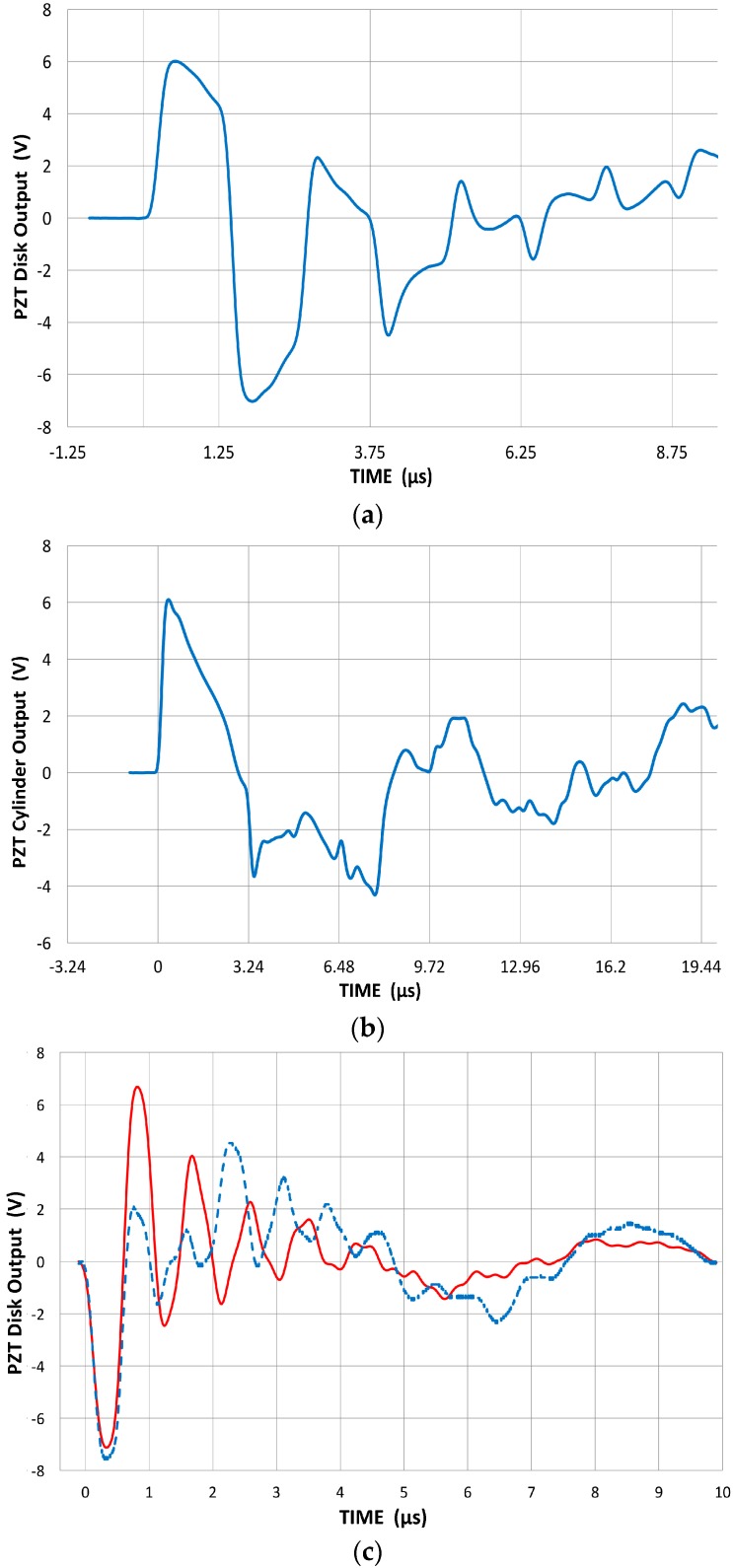
Output signals from PZT disks and cylinder, coupled to V104 reference transmitter. (**a**) Output signals from 400 kHz PZT disk; (**b**) Output signals from 160 kHz PZT cylinder; (**c**) 1 MHz disk with brass backing (blue, dash) and without backing (red).

## References

[1] Ono K. (2016). Calibration Methods of Acoustic Emission Sensors. Materials.

